# Seroprevalence trends of anti-SARS-CoV-2 antibodies and associated risk factors: a population-based study

**DOI:** 10.1007/s15010-023-02011-0

**Published:** 2023-03-04

**Authors:** Stefano Tancredi, Arnaud Chiolero, Cornelia Wagner, Moa Lina Haller, Patricia Chocano-Bedoya, Natalia Ortega, Nicolas Rodondi, Laurent Kaufmann, Elsa Lorthe, Hélène Baysson, Silvia Stringhini, Gisela Michel, Chantal Lüdi, Erika Harju, Irene Frank, Medea Imboden, Melissa Witzig, Dirk Keidel, Nicole Probst-Hensch, Rebecca Amati, Emiliano Albanese, Laurie Corna, Luca Crivelli, Julia Vincentini, Semira Gonseth Nusslé, Murielle Bochud, Valérie D’Acremont, Philipp Kohler, Christian R. Kahlert, Alexia Cusini, Anja Frei, Milo A. Puhan, Marco Geigges, Marco Kaufmann, Jan Fehr, Stéphane Cullati, the Corona Immunitas Research Group, Antonio Amendola, Alexia Anagnostopoulos, Daniela Anker, Anna Maria Annoni, Hélène Aschmann, Andrew Azman, Antoine Bal, Tala Ballouz, Kleona Bezani, Annette Blattmann, Patrick Bleich, Patrick Bodenmann, Peter Buttaroni, Audrey Butty, Anne Linda Camerini, Patricia Orializ Chocano-Bedoya, Prune Collombet, Diana Sofia Da Costa Santos, Agathe Deschamps, Paola D’Ippolito, Anja Domenghino, Richard Dubos, Roxane Dumont, Olivier Duperrex, Julien Dupraz, Malik Egger, Emna El-May, Nacira El Merjani, Nathalie Engler, Adina Mihaela Epure, Lukas Erksam, Sandrine Estoppey, Marta Fadda, Vincent Faivre, Andrea Felappi, Maddalena Fiordelli, Antoine Flahault, Luc Fornerod, Cristina Fragoso Corti, Natalie Francioli, Marion Frangville, Irène Frank, Giovanni Franscella, Clément Graindorge, Idris Guessous, Séverine Harnal, Emilie Jendly, Ayoung Jeong, Laurent Kaiser, Simone Kessler, Christine Krähenbühl, Susi Kriemler, Julien Lamour, Sara Levati, Pierre Lescuyer, Andrea Loizeau, Chantal Luedi, Jean-Luc Magnin, Chantal Martinez, Eric Masserey, Dominik Menges, Rosalba Morese, Nicolai Mösli, Natacha Noël, Daniel Henry Paris, Jérôme Pasquier, Francesco Pennacchio, Stefan Pfister, Giovanni Piumatti, Géraldine Poulain, Caroline Pugin, Milo Puhan, Nick Pullen, Thomas Radtke, Manuela Rasi, Aude Richard, Viviane Richard, Claude-François Robert, Pierre-Yves Rodondi, Serena Sabatini, Khadija Samir, Javier Sanchis Zozaya, Virginie Schlüter, Alexia Schmid, Valentine Schneider, Maria Schüpbach, Nathalie Schwab, Claire Semaani, Alexandre Speierer, Amélie Steiner-Dubuis, Stéphanie Testini, Julien Thabard, Mauro Tonolla, Nicolas Troillet, Agne Ulyte, Sophie Vassaux, Thomas Vermes, Jennifer Villers, Viktor von Wyl, Rylana Wenger, Erin West, Ania Wisniak, María-Eugenia Zaballa, Kyra Zens, Claire Zuppinger

**Affiliations:** 1 Population Health Laboratory (#PopHealthLab), University of Fribourg, Route Des Arsenaux 41, 1700 Fribourg, Switzerland; 2 School of Population and Global Health, McGill University, Montreal, Canada; 3 Institute of Primary Health Care (BIHAM), University of Bern, Bern, Switzerland; 4 Department of General Internal Medicine, Inselspital, Bern University Hospital, University of Bern, Bern, Switzerland; 5 Cantonal Public Health Service of the Canton of Neuchâtel, Neuchâtel, Switzerland; 6 Unit of Population Epidemiology, Division of Primary Care Medicine, Geneva University Hospitals, Geneva, Switzerland; 7 Department of Health and Community Medicine, Faculty of Medicine, University of Geneva, Geneva, Switzerland; 8 University Center for General Medicine and Public Health, University of Lausanne, Lausanne, Switzerland; 9 Department Health Sciences and Medicine, University of Lucerne, Lucerne, Switzerland; 10 Clinical Trial Unit, Lucerne Cantonal Hospital, Lucerne, Switzerland; 11 Swiss Tropical and Public Health Institute, Allschwil, Switzerland; 12 University of Basel, Basel, Switzerland; 13 Institute of Public Health, Faculty of Biomedical Sciences, Università Della Svizzera Italiana, Lugano, Switzerland; 14 Department of Business Economics, Health and Social Care, University of Applied Sciences and Arts of Southern Switzerland, Manno, Switzerland; 15 Center for Primary Care and Public Health (Unisanté), University of Lausanne, Lausanne, Switzerland; 16 Division of Infectious Diseases and Hospital Epidemiology, Cantonal Hospital St Gallen, St Gallen, Switzerland; 17 Department of Infectious Diseases and Hospital Epidemiology, Children’s Hospital of Eastern Switzerland, St. Gallen, Switzerland; 18 Division of Infectious Diseases, Cantonal Hospital of Grisons, Chur, Switzerland; 19 Epidemiology, Biostatistics and Prevention Institute, University of Zurich, Zurich, Switzerland; 20 Department of Readaptation and Geriatrics, University of Geneva, Geneva, Switzerland

**Keywords:** COVID-19 pandemic, SARS-CoV-2, Seroprevalence, Epidemiology, Public health, Surveillance

## Abstract

**Purpose:**

We aimed to assess the seroprevalence trends of SARS-CoV-2 antibodies in several Swiss cantons between May 2020 and September 2021 and investigate risk factors for seropositivity and their changes over time.

**Methods:**

We conducted repeated population-based serological studies in different Swiss regions using a common methodology. We defined three study periods: May–October 2020 (period 1, prior to vaccination), November 2020–mid-May 2021 (period 2, first months of the vaccination campaign), and mid-May–September 2021 (period 3, a large share of the population vaccinated). We measured anti-spike IgG. Participants provided information on sociodemographic and socioeconomic characteristics, health status, and adherence to preventive measures. We estimated seroprevalence with a Bayesian logistic regression model and the association between risk factors and seropositivity with Poisson models.

**Results:**

We included 13,291 participants aged 20 and older from 11 Swiss cantons. Seroprevalence was 3.7% (95% CI 2.1–4.9) in period 1, 16.2% (95% CI 14.4–17.5) in period 2, and 72.0% (95% CI 70.3–73.8) in period 3, with regional variations. In period 1, younger age (20–64) was the only factor associated with higher seropositivity. In period 3, being aged ≥ 65 years, with a high income, retired, overweight or obese or with other comorbidities, was associated with higher seropositivity. These associations disappeared after adjusting for vaccination status. Seropositivity was lower in participants with lower adherence to preventive measures, due to a lower vaccination uptake.

**Conclusions:**

Seroprevalence sharply increased over time, also thanks to vaccination, with some regional variations. After the vaccination campaign, no differences between subgroups were observed.

**Supplementary Information:**

The online version contains supplementary material available at 10.1007/s15010-023-02011-0.

## Introduction

An accurate description of the severe acute respiratory syndrome coronavirus 2 (SARS-CoV-2) spread dynamics is key to informing and driving policymakers’ decisions. Yet, surveillance based on PCR or antigen-reported cases resulted in biased estimates of the virus spread [1] due to a large share of a- or pauci-symptomatic infections [2, 3], changes in care-seeking behaviours, and different screening and diagnostic strategies across regions and over time. For instance, when SARS-CoV-2 first emerged, many European countries had limited testing capacities [4], and some, including Switzerland, restricted testing to patients admitted to hospitals. This led to a surveillance bias [5], with an underestimation of the number of SARS-CoV-2 cases. By contrast, serological studies account for all infections, providing a more representative, albeit less timely, picture of the extent and dynamics of the COVID-19 pandemic.

So far, many SARS-CoV-2 seroprevalence studies have been conducted, both in the general population and in specific subgroups, to monitor the pandemic and inform on population levels of immunity [6–8]. A recent literature review [9] showed substantial worldwide geographical variability in seroprevalence estimates, caused by differences in the extent of infections and vaccination coverage. It also showed evidence of considerable infection under-ascertainment, highlighting the importance of seroprevalence estimates to describe the true number of SARS-CoV-2 infections. However, variabilities in research designs, tests used, or studies quality and reporting, make it challenging to compare estimates between countries or between regions within the same country. In addition to their role in assessing immunity levels and monitoring the virus’s spread, seroepidemiological studies are also a strong tool to understand the drivers of the spread and to identify groups at higher risk of infection. During the pandemic, many factors have been linked to increased seropositivity, including socioeconomic, sociodemographic, or health characteristics. A higher exposure to SARS-CoV-2 is possible in socioeconomically disadvantaged individuals [10] (e.g., with a lower income or lower educational level), and differences in exposure have been found in different age groups [11, 12] or according to job type, health behaviours (e.g., smokers versus non-smokers) or health characteristics (e.g., with respect to different BMI levels or number of comorbidities) [11, 13, 14]. Additionally, the evidence suggests that different levels of stringency of mitigation policies [9] and adherence to preventive measures were also associated with seropositivity [15]. However, countries experienced a wide range of different epidemiological situations; governments recommendations and individual behaviours changed, and vaccines have been rolled out. It is therefore likely that factors associated with seropositivity have also changed over time.

In Switzerland, the Swiss School of Public Health (SSPH +) launched in the early phases of the pandemic the Corona Immunitas research program [16], implementing repeated population-based seroprevalence studies, with the aim of estimating the proportion of the population who developed anti-SARS-CoV-2 antibodies over time. Conducting repeated studies using a common methodology, at regular intervals, and with shared coordination, offers unique strengths to provide a clear picture of the population immunological status over time and across regions, and allows investigating trends in seroprevalence of SARS-CoV-2 antibodies, making comparisons between regions, and investigating differences in the virus’s exposure between different populations’ groups. In light of the above, using data from Corona Immunitas, we aimed to (1) assess the seroprevalence trends of SARS-CoV-2 antibodies in Switzerland between May 2020 and September 2021, both at a quasi-national and cantonal level (descriptive aim), and (2) investigate risk factors for seropositivity and their changes over time (etiologic aim).

## Methods

### Study design

**Fig. 1:**
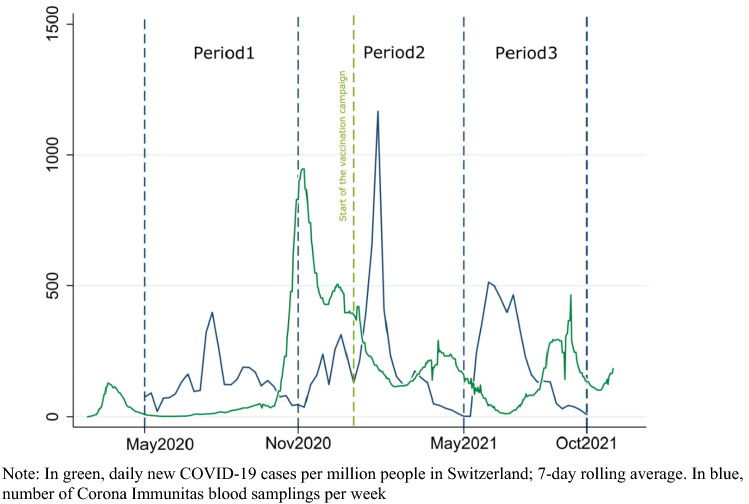
Blood samplings per week and daily confirmed COVID-19 cases reported in Switzerland, May 2020–September 2021

This study is part of Corona Immunitas [16]. Repeated population-based serological studies were conducted in different regions of Switzerland. Testing periods could change for each participating site. Invited participants were randomly selected from the national residential registry by the Swiss Federal Statistical Office for each new assessment wave; 65,500 participants were invited, the average participation rate was around 21%, with regional differences (from 16 to 39%). For this study, we defined three study periods: period 1 from May 2020 to October 2020 (before the launch of the vaccination campaign in Switzerland), period 2 from November 2020 to mid-May 2021 (in the first months of the vaccination campaign), and period 3 from mid-May 2021 to September 2021 (a significant share of the population vaccinated). Each period corresponds to a time window following each of the first three pandemic waves in Switzerland (Fig. 1). This choice was made because estimating seroprevalence after each epidemic wave was deemed more informative for descriptive purposes, and it is in line with the World Health Organization (WHO) recommendations for cross-sectional seroprevalence studies [17]. At each period, participants provided a venous blood sample and filled out a questionnaire on demographic and socioeconomic characteristics, adherence to COVID-19 preventive measures, health status and, once available, vaccination status. The questionnaire could be completed either in person or online (data were collected using REDCap, Research Electronic Data Capture) [18].

### Study population

We included 13,291 participants (in period 1 n = 3402, in period 2 n = 5611, and in period 3 n = 4278) aged 20 and older from 11 Swiss cantons (Additional file1: Fig. S1). Around 5.9 million people live in these cantons, that is roughly 68% of the entire Swiss population. Those aged below 65-years of age and those above were sampled in a ratio of 1:1, with few exceptions in some cantons where only one age group has been recruited. For the second objective of this study, we excluded participants who completed the questionnaire more than 30 days before or after having provided the blood sample for the serology test. The reason for this exclusion was to avoid a possible mismatch between serology results and information reported in the questionnaires (preventive behaviours, health status and socioeconomic status). For the same reason, we also excluded participants who provided information on vaccination status more than 11 days before or after providing the blood sample for the serology test. Figure S2 (Additional file1: Fig. S2) shows a flow diagram of the participants included in the study for each study objective.

### Testing procedure

We analysed venous blood samples using the SenASTrIS assay, developed by the Vaud Central University Hospital (CHUV), the Swiss Federal Institute of Technology in Lausanne (EPFL) and the Swiss Vaccine Center [19]. The assay measures the amount of human immunoglobulins G (IgG) that binds the trimeric SARS-CoV-2 spike protein, induced either by infection or vaccination. The test was validated on a sample of the general population and specificity and sensitivity were 99.7% and 96.6% for the detection of IgG antibodies. Borderline test results (i.e., a signal just below the predefined cut-off) were categorized as seronegative (n = 140, 1%). A detailed description of the test is available elsewhere [19].

### Potential risk factors

For the second objective of this study, we investigated the following potential risk factors, selected based on findings of previous studies, background expert knowledge and a priori reasons for having an increased risk of being seropositive [10, 11, 13, 14, 20–22]: sex, age (20–64 years old vs 65 years and older), educational level (primary, secondary, tertiary), body mass index (BMI; < 18.5; 18.5- 24.9; 25–29.9, ≥ 30 kg/m^2^), household monthly income (≤ 3000 CHF, > 3000–6000 CHF, > 6000–9000 CHF, > 9000 CHF; 1 Euro equalled 1.046 to 1.112 CHF between 1st January 2020 and 25th November 2021), employment status (retired, outside the labour force, self-employed, employed), number of children in the household (none, one child, two or more children), comorbidity score (0, 1, ≥ 2), smoking habit (current smokers vs non-smokers; former smokers were included in the non-smokers category), physical distancing during the previous seven days (frequently, occasionally/rarely), staying at home during the previous seven days (frequently, occasionally/rarely), wearing a mask during the previous seven days (frequently, occasionally/rarely), hygiene measures during the previous seven days (frequently, occasionally/rarely). BMI was categorized according to the World Health Organization standard categories [23]. Educational level was categorized according to the International Standard Classification of Education (ISCED). Physical distancing, staying at home and hygiene measures’ variables were defined as having implemented the measures recommended by public health authorities (e.g.: keeping a distance of 1.5 m, staying at home whenever possible, avoiding unnecessary activities outside the home, no handshaking or hugging, washing hands regularly, sneezing into the elbow, using tissues, etc.). The comorbidity score goes from 0 to ≥ 2 and was calculated using the following possible answers (one point for each disease) to the question “Do you suffer from one or more of the following diseases?”: cancer, immunological diseases, cardiovascular diseases, diabetes, hypertension, respiratory diseases and allergies.

### Statistical analysis

To estimate seroprevalence (objective 1), we used a Bayesian logistic regression model, adjusted for the antibody test sensitivity and specificity performances [24]. Seroprevalence estimates were weighted by the age and sex distribution of the population of each canton. We investigated the association between potential risk factors and seropositivity (objective 2) using Poisson regression models and expressed as prevalence ratios (PR) and 95% confidence intervals. Robust variance estimators were used to relax the assumption that the outcome distribution followed a Poisson distribution. Sex, age, educational level, BMI, income, employment status, number of children in the household, comorbidity score and smoking habit were included in the models (hereafter, model 1). Results were stratified by study period. Models for period 3 were adjusted for vaccination status (hereafter, model 2; in Switzerland the vaccination campaign started at the end of December 2020, during the second period of this study). To investigate seropositivity risk factors and their changes over time (objective 2), we used multiple imputation by chained equations to impute any missing data (30 imputations). Statistical analyses were conducted using Stata 17 software (Stata Corp, TX, 2021) and R Statistical Software (version 4.1.2; R Foundation for Statistical Computing, Vienna, Austria).

We also performed several sensitivity analyses: (1) including participants who had completed the questionnaire on demographic and socioeconomic characteristics, adherence to COVID-19 preventive measures, and health status, more than 60 days before and after their blood sample; (2) including a third age category (from 20 to 34 years old; based on the hypothesis that people in this category could have had more social interactions and therefore an increased risk of being infected) and (3) using a score computed from the preventive behaviours variables (hereafter, preventive behaviours score). The score goes from 0 to 4; one point for every “occasionally/rarely” answer to the questions on preventive behaviours. The higher the score, the less frequent the adherence to preventive behaviours.

## Results

### Characteristics of the sample

We included 13,291 respondents (53% females), with a mean age of 55.9 years (SD = 16.9). Characteristics of the participants are summarized in Table 1. Participants’ characteristics across study periods and by cantons are detailed in Tables S1 and S2 (Additional file 1: Tables S1 and S2). Some 61% of participants were aged between 20 and 64 years and 39% were 65 years and older. 46% of participants were highly educated, 42% were employed and 23% lived with children. 47% had one or more comorbidities. In our sample people aged over 65 years were overrepresented by design, and smokers, employed participants, households with one or more than one child, low-income households and people with only primary education were slightly underrepresented [25].

**Table 1:** Characteristics of participants (n = 13,291), Corona Immunitas study, Switzerland, May 2020—September 2021

Sociodemographic characteristics	
Sex	
Female	53%
Male	47%
Age group	
≥ 65	39%
20–64	61%
Children in the household	
No children	77%
One child	9%
More than one child	14%
Socioeconomic characteristics	
Educational level^a^	
Tertiary	46%
Secondary	48%
Primary	6%
Household income	
> CHF 9000	34%
CHF > 6000–9000	28%
CHF > 3000–6000	28%
CHF ≤ 3000	10%
Employment status	
Retired	38%
Outside the labour force^b^	10%
Self employed	10%
Employed	42%
Health status	
Body Mass Index	
< 18.5	3%
18.5–24.9	52%
25–29.9	33%
≥ 30	12%
Comorbiditiy score^c^	
0	53%
1	32%
≥ 2	15%
Smoking	
Non-smoker	84%
Smoker	16%
Preventive behaviours	
Physical distancing during previous 7 days	
Frequently	91%
Occasionally/rarely	9%
Staying at home during previous 7 days	
Frequently	69%
Occasionally/rarely	31%
Wearing mask during previous 7 days	
Frequently	83%
Occasionally/rarely	17%
Hygiene measures during previous 7 days	
Frequently	94%
Occasionally/rarely	6%

^a^International Standard Classification of Education (ISCED)

^b^Outside the labour force includes participants in training/studying and unemployed participants

^c^Comorbidity score goes from 0 to ≥ 2 and was calculated using the following possible answers: cancer; immunological diseases; cardiovascular diseases or diabetes or hypertension; respiratory diseases; allergies

### Seroprevalence estimates and trends

**Fig. 2:**
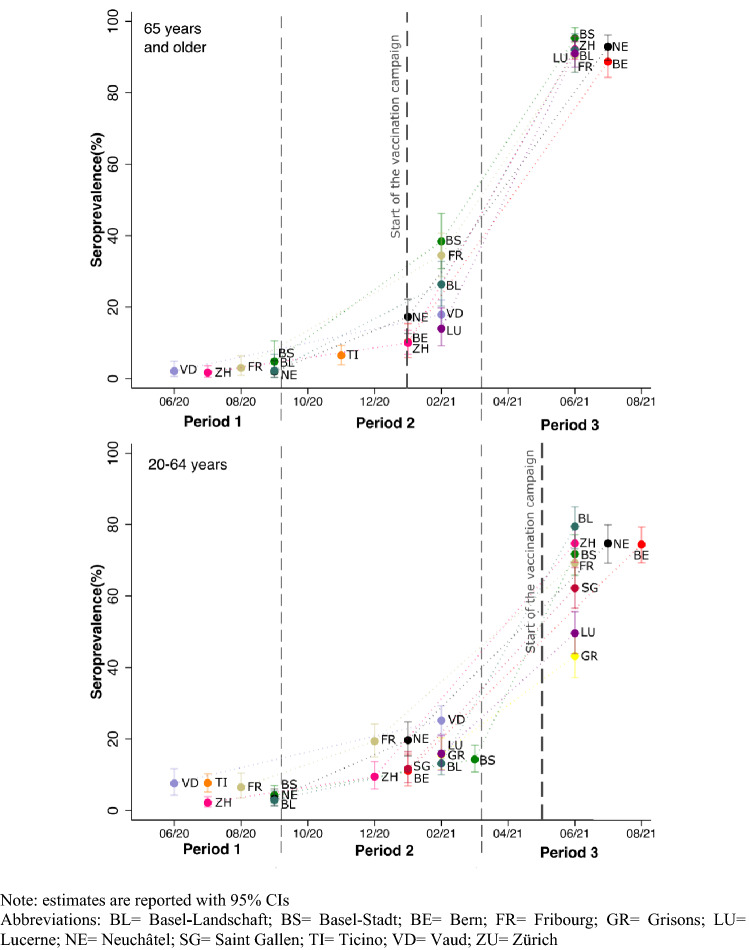
Trends of seroprevalence estimates (IgG anti SARS-CoV-2 Spike) per canton and by age group, Corona Immunitas study, Switzerland, May 2020–September 2021

During period 1, seroprevalence was 3.7% (95% CI 2.1–4.9). It increased to 16.2% (95% CI 14.4–17.5) during period 2 and to 72.0% (95% CI 70.3–73.8) during period 3. Seroprevalence varied by age group, with higher estimates in younger participants (20–64 years) during period 1 and in older participants (65 years and older) during period 3 (Additional file 1: Table S3). There were some regional variations between cantons (Table 2 and Fig. 2). During period 1, seroprevalence in cantons from the French and Italian speaking regions of Switzerland ranged from 3.0 to 7.7%, and in cantons from the German speaking regions from 2.1 to 5.0%. We found substantial differences between cantons during periods 2 and 3.

**Table 2:** Seroprevalence estimates^a^ (IgG anti Sars-CoV-2 Spike) by study period and canton, Corona Immunitas study, Switzerland, May 2020–September 2021

Time window	Period 1, n = 3402	Period 2, n = 5611	Period 3, n = 4278
	01/05/2020–31/10/2020	01/11/2020–15/05/2021	16/05/2021–31/09/2021
	% (95%CI)	% (95%CI)	% (95%CI)
National level	3.7 (2.1–4.9)	16.2 (14.4–17.5)	72.0 (70.3–73.8)
Basel-Landschaft	2.9 (1.3–5.4)	16.5 (13.4–19.9)	82.8 (78.0–87.3)
Basel-Stadt	5.0 (2.6–7.8)	19.7 (16.2–23.2)	77.2 (72.7–81.6)
Bern	NA	10.9 (7.4–14.7)	78.1 (74.1–82.0)
Fribourg	5.9 (3.2–9.1)	22.5 (18.7–26.6)	73.5 (68.4–78.4)
Grisons^b^	NA	15.7 (11.5–20.3)	43.2 (37.2–49.3)
Lucerne	NA	15.5 (11.7–19.7)	58.9 (54.4–63.6)
Neuchâtel	3.0 (1.4–5.4)	19.2 (15.4–23.1)	79.2 (74.8–83.2)
Saint Gallen^b^	NA	11.7 (7.8–16.5)	62.2 (56.6–68.0)
Ticino^c^	7.7 (5.3–10.3)	6.8 (4.1–9.7)	NA
Vaud	6.5 (3.8–9.8)	23.7 (20.3–27.1)	NA
Zurich	2.1 (1.0–3.6)	9.7 (6.7–13.0)	78.5 (74.3–82.5)

Samplings in Bern, Grisons, Lucerne and Saint Gallen started after period 1. Data for Ticino and Vaud period 3 were not available for the analyses presented in this study

*IgG* immunoglobulin G, *NA* not available

^a^Seroprevalence was estimated using Bayesian regression adjusted for the antibody test sensitivity and specificity performances and weighted by age and sex of the general population of each canton

^b^In cantons Grisons and Saint Gallen, only participants aged from 20 to 64 years were tested

^c^In canton Ticino, during period 1, only participants aged from 20 to 64 were tested. During period 2 only data from people aged 65 years or more were available for these analyses

### Factors associated with seropositivity

Table 3 shows the results of the multivariable models by study period. Information on missing data are reported in Tables S4 and S5 (Additional file1: Tables S4 and S5). Before the start of the vaccination campaign (period 1), participants aged between 20 and 64 years had a higher prevalence of seropositivity (PR = 2.32, 95% CI 1.03–5.22) compared to older participants. After the start of the vaccination campaign (period 3), participants aged 20–64 years old (PR = 0.85, 95% CI 0.78–0.93), with a low household income (PR = 0.75, 95% CI 0.68–0.82) or with an employment status different from retired had a lower prevalence of seropositivity compared to reference categories. Participants with a BMI of 25 or more (PR = 1.12, 95% CI 1.04–1.19) or with one or more comorbidities (PR = 1.12, 95% CI 1.06–1.18) had a higher prevalence of seropositivity. However, all these differences disappeared upon adjustment for vaccination status in period 3 (percentages of vaccinated participants in period 3 are reported in additional file 1: Table S6).

**Table 3:** Association of sociodemographic, socioeconomic characteristics and health status with SARS-CoV-2 seropositivity across study periods, Corona Immunitas study, Switzerland, May 2020–September 2021

Factor	Period 1, n = 3108 (01/05/2020–31/10/2020)	Period 2, n = 4969 (01/11/2020–15/05/2021)	Period 3, n = 2836 (16/05/2021–31/09/2021)	
	Model 1^a^, PR (95%)	Model 1^a^, PR (95%)	Model 1^a^, PR (95%)	Model 2^b^, PR (95%)
Sex				
Female	1 [Reference]	1 [Reference]	1 [Reference]	1 [Reference]
Male	1.15 (0.82–1.62)	1.05 (0.92–1.20)	0.93 (0.89–0.98)	0.97 (0.94–1.00)
Age groups				
≥ 65	1 [Reference]	1 [Reference]	1 [Reference]	1 [Reference]
20–64	2.32 (1.03–5.22)	1.22 (0.96 -1.54)	0.85 (0.78–0.93)	0.94 (0.89–0.99)
Children in the household				
No children	1 [Reference]	1 [Reference]	1 [Reference]	1 [Reference]
One child	0.71 (0.40–1.24)	1.20 (0.96–1.49)	0.90 (0.82–1.00)	0.97 (0.90–1.04)
More than one child	1.01 (0.64–1.60)	1.11 (0.90–1.35)	0.92 (0.85–0.99)	1.06 (0.99–1.12)
Educational level				
Tertiary	1 [Reference]	1 [Reference]	1 [Reference]	1 [Reference]
Secondary	1.62 (1.12–2.35)	0.83 (0.72–0.96)	0.95 (0.91–0.99)	1.01 (0.98–1.04)
Primary	1.49 (0.59–3.79)	1.15 (0.89–1.50)	0.92 (0.82–1.03)	1.01 (0.93–1.10)
Household income				
> CHF 9000	1 [Reference]	1 [Reference]	1 [Reference]	1 [Reference]
CHF > 6000–9000	0.72 (0.44–1.19)	1.07 (0.90–1.27)	0.88 (0.83–0.93)	0.99 (0.95–1.03)
CHF > 3000–6000	0.76 (0.46–1.27)	1.25 (1.04–1.51)	0.83 (0.78–0.88)	0.97 (0.94–1.01)
CHF ≤ 3000	0.79 (0.38–1.65)	0.94 (0.71–1.26)	0.75 (0.68–0.82)	0.94 (0.89–1.01)
Employment status				
Retired	1 [Reference]	1 [Reference]	1 [Reference]	1 [Reference]
Outside the labour force^b^	1.09 (0.46–2.60)	0.69 (0.54–0.89)	0.84 (0.72–0.97)	1.00 (0.90–1.10)
Self employed	1.10 (0.50–2.41)	0.68 (0.49–0.95)	0.78 (0.69–0.88)	0.97 (0.90–1.06)
Employed	0.56 (0.25–1.26)	0.81 (0.63–1.04)	0.84 (0.77–0.93)	1.00 (0.95–1.06)
Body Mass Index				
18.5–24.9	1 [Reference]	1 [Reference]	1 [Reference]	1 [Reference]
< 18.5	0.49 (0.12–1.96)	1.07 (0.70–1.63)	0.99(0.86–1.14)	1.04 (0.93–1.16)
25–29.9	0.72 (0.49–1.07)	1.12 (0.97–1.30)	1.08(1.03–1.13)	1.04 (1.00–1.07)
≥ 30	0.67 (0.38–1.17)	1.18 (0.97–1.44)	1.12(1.04–1.19)	1.04 (1.00–1.09)
Comorbiditiy score^d^				
0	1 [Reference]	1 [Reference]	1 [Reference]	1 [Reference]
1	1.23 (0.82–1.79)	0.95 (0.82–1.10)	1.08(1.03–1.13)	1.01 (0.98–1.04)
≥ 2	1.26 (0.74–2.16)	1.24 (1.04–1.48)	1.12(1.06–1.18)	1.00 (0.97–1.03)
Smoking				
Non-smoker	1 [Reference]	1 [Reference]	1 [Reference]	1 [Reference]
Smoker	0.80 (0.49–1.29)	0.81 (0.66–0.99)	0.93 (0.86–1.00)	0.97 (0.92–1.01)

^a^Model adjusted for: sex, age, educational level, body mass index, income, employment status, children in the household, comorbidity score and smoking habit

^b^Model additionally adjusted for vaccination status

^c^Outside the labour force includes participants in training/studying and not employed participants

^d^Comorbidity score goes from 0 to ≥ 2 and was calculated using the following possible answers: cancer; immunological diseases; cardiovascular diseases or diabetes or hypertension; respiratory diseases; allergies

None of the self-reported preventive behaviours (Table 4) were associated with seropositivity before the start of the vaccination campaign (period 1). In period 3, participants who reported to occasionally or rarely practice physical distancing (PR = 0.81, 95% CI 0.75–0.89), stay at home (PR = 0.94, 95% CI 0.90–0.98), wear a mask (PR = 0.76, 95% CI 0.70–0.84) and perform hygiene measures (PR = 0.79, 95% CI 0.72–0.87) had a lower prevalence of seropositivity compared to participants who frequently adhered to preventive behaviours. All these differences disappeared after adjusting for vaccination status.

**Table 4:** Association of recommended preventive behaviours with SARS-CoV-2 seropositivity across study periods, Corona Immunitas study, Switzerland, May 2020–September 2021

Factor	Period 1, n = 2151 (01/05/2020–31/10/2020)	Period 2, n = 4969 (01/11/2020–15/05/2021)	Period 3, n = 2836 (16/05/2021–31/09/2021)	
	Model 1^a^, PR (95%)	Model 1^a^, PR (95%)	Model 1^a^, PR (95%)	Model 2^b^, PR (95%)
Physical distancing during previous 7 days				
Frequently	1 [Reference]	1 [Reference]	1 [Reference]	1 [Reference]
Occasionally/rarely	1.25 (0.61–2.59)	1.52 (1.18–1.96)	0.81 (0.75–0.89)	0.97 (0.92–1.02)
Staying at home during previous 7 days				
Frequently	1 [Reference]	1 [Reference]	1 [Reference]	1 [Reference]
Occasionally/rarely	1.43 (0.86–2.37)	1.13 (0.96–1.33)	0.94 (0.90–0.98)	1.02 (0.99–1.05)
Wearing mask during previous 7 days				
Frequently	1 [Reference]	1 [Reference]	1 [Reference]	1 [Reference]
Occasionally/rarely	0.68 (0.41–1.13)	1.06 (0.78–1.43)	0.76 (0.70–0.84)	0.96 (0.91–1.02)
Hygiene measures during previous 7 days				
Frequently	1 [Reference]	1 [Reference]	1 [Reference]	1 [Reference]
Occasionally/rarely	0.97 (0.31–3.02)	0.88 (0.62–1.25)	0.79 (0.72–0 .87)	1.01 (0.94–1.08)

Data from Ticino and data from Vaud period 1 were not included because not harmonizable with data from other sites

^a^Model adjusted for sex, age, educational level, body mass index, income, employment status, children in the household, comorbidity score and smoking habit

^b^Model additionally adjusted for vaccination status

Sensitivity analyses gave similar results as the main analyses and results are shown in supplementary material (Additional file1: Tables S7–S10).

## Discussion

### Main findings

Seroprevalence in Switzerland rose sharply between May 2020 and September 2021, with some regional variations, from 3.7% (95% CI 2.1–4.9) in May–October 2020, to 16.2% (95% CI 14.4–17.5) between November 2020 and mid-May 2021, and finally 72.0% (95% CI 70.3–73.8) between mid-May and September 2021. Before the start of the vaccination campaign, seropositivity differed by age but not by other factors. After the start of the vaccination campaign, seropositivity was higher among participants over 65 years, with a high income, retired, overweight or obese, or with other comorbidities, due to a higher vaccination uptake. Seropositivity was lower in participants with lower adherence to preventive measures, due to a reduced propensity for vaccination uptake.

### Comparison with other studies

This study’s findings describe the evolution of the SARS-CoV-2 spread and of population immunological status in the first phases of the COVID-19 pandemic in several cantons of Switzerland, accounting for under-ascertainment and differences in testing strategies across Swiss cantons. European seroprevalence estimates varied widely during the pandemic, depending on study populations, study periods and methods used. However, our seroprevalence estimates were roughly similar to estimates found by other seroprevalence surveys in the same periods in other Swiss cantons [24, 26] and to pooled estimates from other European high-income countries [9]. We found some variations in seroprevalence estimates between cantons during period 1, with estimates ranging from 3.0 to 6.5% in the French speaking cantons, being 7.7% in Ticino, and ranging from 2.1 to 5.0% in German speaking cantons. These results are particularly interesting in light of the fact that, especially at the beginning of the pandemic, comparisons between studies were hindered by differences in study designs. We also found substantial differences during period 2 and 3. However, these results are difficult to interpret, since seroprevalence estimates were strongly influenced by vaccination rates during these periods, and differences in testing periods could have resulted in different estimates.

During the first period of this study, i.e., before the start of the vaccination campaign, we found a higher prevalence of seropositivity in participants aged between 20 and 64 years compared to those aged 65 years and older. Other studies showed higher seroprevalence in younger adults [10–13] compared to older population’s groups, and this could be due to the fact that younger populations were considered at lower risk of severe illness and therefore could have had more social interactions. No other factor was associated with seropositivity during the first period of this study, despite several studies showing differences in seropositivity according to socioeconomic characteristics (e.g., higher seroprevalence in people with lower income or lower educational level) [10], health behaviours (e.g., higher seroprevalence in smokers vs non- smokers) [11, 13] or other sociodemographic characteristics (e.g., higher seroprevalence in households with more than one child) [13]. The higher prevalence of seropositivity found during the third period of this study among participants aged over 65 years, overweight or obese, retired and with other comorbidities, was due to a higher vaccination rate in these subgroups. These results were expected, since the vaccination campaign in Switzerland prioritised people with a higher risk of severe illness and death (i.e., older people and people with comorbidities or a high BMI) [27]. Having a high household income was also associated with higher seropositivity due to a higher vaccination uptake. This finding is consistent with other studies conducted in Switzerland [28] and elsewhere [29, 30].

Regarding preventive behaviours, despite several personal and social preventive measures associated with a reduction in the incidence of COVID-19 [21, 31], we did not find associations between adherence to preventive behaviours and seropositivity before the start of the vaccination campaign (period 1). This result could be due to selection bias, as people who adhered less to preventive measures were also less likely to participate in this study. Another hypothetical explanation is that people who did not frequently adhere to the recommended measures benefited from the collective adherence to those same measures, or from the low seroprevalence in period 1. During the last study period, we found a lower prevalence of seropositivity in people with lower adherence to recommended preventive behaviours, especially in participants who less frequently wore masks. This was explained by a lower vaccination uptake in these groups. Other studies investigated the associations between willingness to receive the COVID-19 vaccine and adherence to preventive behaviours [32], showing that people who are more prone to follow prevention recommendations are also more likely to get vaccinated. Overall, the associations between risk factors and seroprevalence during period 2 were difficult to interpret because period 2 included blood sample collected both before and after the vaccination campaign and because, during the first months of the vaccination campaign, self-reported information on vaccination status was less reliable, due to organizational difficulties in promptly modifying the questionnaires to include questions on vaccination status.

### Strengths and limitations

This study has some limitations. Overall, the participation rate was moderate (21%). Moreover, despite random representative samples of the population being invited, selection bias is possible, with a higher participation rate of highly educated participants compared to the Swiss general population. Further, seroprevalence could be underestimated due to waning immunity [33], people failing to produce antibodies [34] and due to the fact that we only measured the amount of anti-SARS-CoV-2 IgGs, without assessing other types of antibodies. We were therefore not able to distinguish between infection-related and vaccination-related antibodies. Information bias is also possible, since the information collected through the questionnaire was self-reported. The key strengths of our study include the use of a large population-based sample covering a significant proportion of the country and with repeated samplings over time, the use of a previously validated test with high sensitivity and specificity, and post-stratification weights to account for differences in sex and age.

## Conclusions

Seroprevalence in Switzerland has increased sharply over time, also thanks to the increasing vaccination coverage, with some regional differences. After the vaccination campaign, no differences between subgroups were observed.

### Supplementary Information

Below is the link to the electronic supplementary material.

Supplementary file1 (DOCX 321 KB)

## Data Availability

Deidentified individual participant data underlying the findings of this study will be available for researchers submitting a methodologically sound proposal to achieve the aims of the proposal after the publication of this article. Access to data requires contacting Corona Immunitas.

## References

[1] Byambasuren O, Dobler CC, Bell K, Rojas DP, Clark J, McLaws ML (2021). Comparison of seroprevalence of SARS-CoV-2 infections with cumulative and imputed COVID-19 cases: systematic review. PLoS One.

[2] Sah P, Fitzpatrick MC, Zimmer CF, Abdollahi E, Juden-Kelly L, Moghadas SM, et al. Asymptomatic SARS-CoV-2 infection: a systematic review and meta-analysis. Proc Natl Acad Sci USA. 2021;118(34):e2109229118 10.1073/pnas.2109229118 PMC8403749 34376550

[3] Alene M, Yismaw L, Assemie MA, Ketema DB, Mengist B, Kassie B (2021). Magnitude of asymptomatic COVID-19 cases throughout the course of infection: a systematic review and meta-analysis. PLoS One.

[4] Sharfstein JM, Becker SJ, Mello MM (2020). Diagnostic testing for the novel coronavirus. JAMA.

[5] Tancredi S, Anker D, Rosella L, Chiolero A. Elimination of covid-19: beware of surveillance bias. Bmj. 2021;374:n2126. 10.1136/bmj.n2126 34479862

[6] Bobrovitz N, Arora RK, Cao C, Boucher E, Liu M, Donnici C (2021). Global seroprevalence of SARS-CoV-2 antibodies: a systematic review and meta-analysis. PLoS One.

[7] Rostami A, Sepidarkish M, Fazlzadeh A, Mokdad AH, Sattarnezhad A, Esfandyari S (2021). Update on SARS-CoV-2 seroprevalence: regional and worldwide. Clin Microbiol Infect.

[8] Galanis P, Vraka I, Fragkou D, Bilali A, Kaitelidou D (2021). Seroprevalence of SARS-CoV-2 antibodies and associated factors in healthcare workers: a systematic review and meta-analysis. J Hosp Infect.

[9] Bergeri I, Whelan M, Ware H, Subissi L, Nardone A, Lewis HC, et al. Global SARS-CoV-2 seroprevalence: a systematic review and meta-analysis of standardized population-based studies from Jan 2020-May 2022. medRxiv. 2022:2021.12.14.21267791. 10.1371/journal.pmed.1004107 PMC9648705 36355774

[10] Basto-Abreu A, Carnalla M, Torres-Ibarra L, Romero-Martínez M, Martínez-Barnetche J, López-Martínez I (2022). Nationally representative SARS-CoV-2 antibody prevalence estimates after the first epidemic wave in Mexico. Nat Commun.

[11] Richard A, Wisniak A, Perez-Saez J, Garrison-Desany H, Petrovic D, Piumatti G (2022). Seroprevalence of anti-SARS-CoV-2 IgG antibodies, risk factors for infection and associated symptoms in Geneva, Switzerland: a population-based study. Scand J Public Health.

[12] Vos ERA, den Hartog G, Schepp RM, Kaaijk P, van Vliet J, Helm K (2021). Nationwide seroprevalence of SARS-CoV-2 and identification of risk factors in the general population of the Netherlands during the first epidemic wave. J Epidemiol Community Health.

[13] Carrat F, de Lamballerie X, Rahib D, Blanché H, Lapidus N, Artaud F (2021). Antibody status and cumulative incidence of SARS-CoV-2 infection among adults in three regions of France following the first lockdown and associated risk factors: a multicohort study. Int J Epidemiol.

[14] Beaumont A, Durand C, Ledrans M, Schwoebel V, Noel H, Le Strat Y (2021). Seroprevalence of anti-SARS-CoV-2 antibodies after the first wave of the COVID-19 pandemic in a vulnerable population in France: a cross-sectional study. BMJ Open.

[15] Abaluck J, Kwong LH, Styczynski A, Haque A, Kabir MA, Bates-Jefferys E (2022). Impact of community masking on COVID-19: a cluster-randomized trial in Bangladesh. Science.

[16] West EA, Anker D, Amati R, Richard A, Wisniak A, Butty A (2020). Corona Immunitas: study protocol of a nationwide program of SARS-CoV-2 seroprevalence and seroepidemiologic studies in Switzerland. Int J Public Health.

[17] World Health O. Population-based age-stratified seroepidemiological investigation protocol for COVID-19 virus infection, 17 March 2020. Geneva: World Health Organization; 2020 2020. Contract No.: WHO/2019-nCoV/Seroepidemiology/2020.1.

[18] Harris PA, Taylor R, Thielke R, Payne J, Gonzalez N, Conde JG (2009). Research electronic data capture (REDCap)–a metadata-driven methodology and workflow process for providing translational research informatics support. J Biomed Inform.

[19] Fenwick C, Croxatto A, Coste AT, Pojer F, André C, Pellaton C (2021). Changes in SARS-CoV-2 Spike versus nucleoprotein antibody responses impact the estimates of infections in population-based seroprevalence studies. J Virol..

[20] Galmiche S, Charmet T, Schaeffer L, Paireau J, Grant R, Chény O (2021). Exposures associated with SARS-CoV-2 infection in France: a nationwide online case-control study. Lancet Reg Health Eur.

[21] Talic S, Shah S, Wild H, Gasevic D, Maharaj A, Ademi Z (2021). Effectiveness of public health measures in reducing the incidence of covid-19, SARS-CoV-2 transmission, and covid-19 mortality: systematic review and meta-analysis. BMJ.

[22] Blankenberger J, Kaufmann M, Albanese E, Amati R, Anker D, Camerini AL (2022). Is living in a household with children associated with SARS-CoV-2 seropositivity in adults? Results from the Swiss national seroprevalence study Corona Immunitas. BMC Med.

[23] World Health Organization. WHO global infoBase team. The SuRF Report 2. Surveillance of chronic disease Risk Factors: Country-level data and comparable estimates. Geneva; 2005.

[24] Stringhini S, Wisniak A, Piumatti G, Azman AS, Lauer SA, Baysson H (2020). Seroprevalence of anti-SARS-CoV-2 IgG antibodies in Geneva, Switzerland (SEROCoV-POP): a population-based study. Lancet.

[25] Federal Statistical Office (FSO). Available from: https://www.bfs.admin.ch/bfs/en/home/statistics.html.

[26] Stringhini S, Zaballa ME, Pullen N, Perez-Saez J, de Mestral C, Loizeau AJ (2021). Seroprevalence of anti-SARS-CoV-2 antibodies 6 months into the vaccination campaign in Geneva, Switzerland, 1 June to 7 July 2021. Euro Surveill.

[27] Federal Office of Public Health. Available from: https://www.bfs.admin.ch/bfs/en/home/statistics.html.

[28] Heiniger S, Schliek M, Moser A, von Wyl V, Höglinger M (2022). Differences in COVID-19 vaccination uptake in the first 12 months of vaccine availability in Switzerland—a prospective cohort study. Swiss Med Wkly.

[29] Bayati M, Noroozi R, Ghanbari-Jahromi M, Jalali FS (2022). Inequality in the distribution of Covid-19 vaccine: a systematic review. Int J Equity Health.

[30] Williams AM, Clayton HB, Singleton JA (2022). Racial and ethnic disparities in COVID-19 vaccination coverage: the contribution of socioeconomic and demographic factors. Am J Prev Med.

[31] Iezadi S, Gholipour K, Azami-Aghdash S, Ghiasi A, Rezapour A, Pourasghari H (2021). Effectiveness of non-pharmaceutical public health interventions against COVID-19: a systematic review and meta-analysis. PLoS One.

[32] Lam CN, Kaplan C, Saluja S (2022). Relationship between mask wearing, testing, and vaccine willingness among Los Angeles County adults during the peak of the COVID-19 pandemic. Transl Behav Med.

[33] Gaebler C, Wang Z, Lorenzi JCC, Muecksch F, Finkin S, Tokuyama M (2021). Evolution of antibody immunity to SARS-CoV-2. Nature.

[34] Liu W, Russell RM, Bibollet-Ruche F, Skelly AN, Sherrill-Mix S, Freeman DA (2021). Predictors of nonseroconversion after SARS-CoV-2 infection. Emerg Infect Dis.

