# Supplementary material for: Seroprevalence trends of anti-SARS-CoV-2 antibodies and associated risk factors: a population-based study
Source: Infection. 2023 Mar 4;51(5):1453–65. doi: 10.1007/s15010-023-02011-0 (PMC9985433; doi:10.1007/s15010-023-02011-0)
Supplement: Supplementary file 1 — Supplementary file1 (DOCX 321 KB) [file 15010_2023_2011_MOESM1_ESM.docx]

**Seroprevalence trends of anti-SARS-CoV-2 antibodies and associated risk factors: a population-based study**

**Submitted to “Infection”**

Stefano Tancredi ^1^, Arnaud Chiolero ^1,2,3^, Cornelia Wagner ^1^, Moa Lina Haller ^3,4^, Patricia O. Chocano Bedoya ^1,3^, Natalia Ortega ^1,3^, Nicolas Rodondi ^3,4^, Laurent Kaufmann ^5^, Elsa Lorthe ^6^, Hélène Baysson ^6,7^, Silvia Stringhini ^6,7,8^, Gisela Michel ^9^, Chantal Lüdi ^9^, Erika Harju ^9,10^, Irene Frank ^10^, Medea Imboden ^11,12^, Melissa Witzig ^11,12^, Dirk Keidel ^11,12^, Nicole Probst-Hensch ^11,12^, Rebecca Amati ^13^, Emiliano Albanese ^13^, Laurie Corna ^14^, Luca Crivelli ^14^, Julia Vincentini ^15^, Semira Gonseth Nusslé ^15^, Murielle Bochud ^15^, Valérie D’Acremont ^11,15^, Philipp Kohler ^16^, Christian R. Kahlert ^16,17^, Alexia Cusini ^18^, Anja Frei ^19^, Milo A. Puhan ^19^, Marco Geigges ^19^, Marco Kaufmann ^19^, Jan Fehr ^19^, Stéphane Cullati ^1,20^, on behalf of the Corona Immunitas Research Group

1. Population Health Laboratory (#PopHealthLab), University of Fribourg, Fribourg, Switzerland
2. School of Population and Global Health, McGill University, Montreal, Canada.
3. Institute of Primary Health Care (BIHAM), University of Bern, Bern, Switzerland
4. Department of General Internal Medicine, Inselspital, Bern University Hospital, University of Bern, Switzerland
5. Cantonal Public Health Service of the Canton of Neuchâtel, Neuchâtel, Switzerland
6. Unit of Population Epidemiology, Division of Primary Care Medicine, Geneva University Hospitals, Geneva, Switzerland
7. Department of Health and Community Medicine, Faculty of Medicine, University of Geneva, Geneva, Switzerland
8. University Center for General Medicine and Public Health, University of Lausanne, Lausanne, Switzerland
9. Department Health Sciences and Medicine, University of Lucerne, Lucerne, Switzerland
10. Clinical Trial Unit, Lucerne Cantonal Hospital, Lucerne, Switzerland
11. Swiss Tropical and Public Health Institute, Allschwil, Switzerland
12. University of Basel, Basel, Switzerland
13. Institute of Public Health, Faculty of Biomedical Sciences, Università della Svizzera Italiana, Lugano, Switzerland
14. Department of Business Economics, Health and Social Care at the University of Applied Sciences and Arts of Southern Switzerland, Manno, Switzerland
15. Center for Primary Care and Public Health (Unisanté), University of Lausanne, Switzerland
16. Cantonal Hospital St Gallen, Division of Infectious Diseases and Hospital Epidemiology, St Gallen, Switzerland
17. Children's Hospital of Eastern Switzerland, Department of Infectious Diseases and Hospital Epidemiology, St. Gallen, Switzerland
18. Cantonal Hospital of Grisons, Division of Infectious Diseases, Chur, Switzerland
19. Epidemiology, Biostatistics and Prevention Institute, University of Zurich, Zurich, Switzerland
20. Department of Readaptation and Geriatrics, University of Geneva, Geneva, Switzerland

**CORRESPONDING AUTHOR**

Stefano Tancredi, Population Health Laboratory (#PopHealthLab), University of Fribourg, Route des Arsenaux 41, CH - 1700 Fribourg, Switzerland, [stefano.tancredi@unifr.ch](mailto:stefano.tancredi@unifr.ch) [,+41 26 300 9492](tel:+41%2026%20300%209492)

**Supplementary Information**

Contents

[Figure S1. Geographic location of the population-based seroprevalence studies used for this analysis, Corona Immunitas study, Switzerland, May 2020 - September 2021 2](#_Toc118377570)

[Figure S2. Flow chart of participants included in the analysis of seroprevalence trends (objective 1) and in the analysis for risk factors (objective 2), Corona Immunitas study, Switzerland, May 2020 - September 2021 3](#_Toc118377571)

[Table S1: Characteristics of participants across study periods, Corona Immunitas study, Switzerland, May 2020 – September 2021 4](#_Toc118377572)

[Table S2: Characteristics of participants by canton, Corona Immunitas study, Switzerland, May 2020 – September 2021 6](#_Toc118377573)

[Table S3: Weighted seroprevalence (IgG antispike) by study period, stratified by sex and age, Corona Immunitas study, Switzerland, May 2020 – September 2021 15](#_Toc118377574)

[Table S4: Missing data for sociodemographic, socioeconomic and health status variables by study period (objective 2), Corona Immunitas study, Switzerland, May 2020 – September 2021 16](#_Toc118377575)

[Table S5: Missing data for preventive behaviours variables by study period (objective 2), Corona Immunitas study, Switzerland, May 2020 – September 2021 16](#_Toc118377576)

[Table S6: percentages of vaccinated participants (period 3, objective 2), Corona Immunitas study, Switzerland 17](#_Toc118377577)

[Table S7: Association of sociodemographic, socioeconomic factors and health status with SARS-CoV-2 seropositivity across study periods; sensitivity analysis including participants who replied to the questionnaire more than 60 days before and after their blood sample, Corona Immunitas study, Switzerland, May 2020 – September 2021 18](#_Toc118377578)

[Table S8: Association of recommended preventive behaviours with SARS-CoV-2 seropositivity across study periods, sensitivity analysis including participants who reply to the questionnaire more than 60 days before and after their blood sample, Corona Immunitas study, Switzerland, May 2020 – September 2021 20](#_Toc118377579)

[Table S9: Sensitivity analysis including 20-34 age category, Corona Immunitas study, Switzerland, May 2020 – September 2021 21](#_Toc118377580)

[Table S10: Sensitivity analysis including preventive behaviours score, Corona Immunitas study, Switzerland, May 2020 – September 2021 21](#_Toc118377581)

**Figure S1. Geographic location of the population-based seroprevalence studies used for this analysis, Corona Immunitas study, Switzerland, May 2020 - September 2021**

**
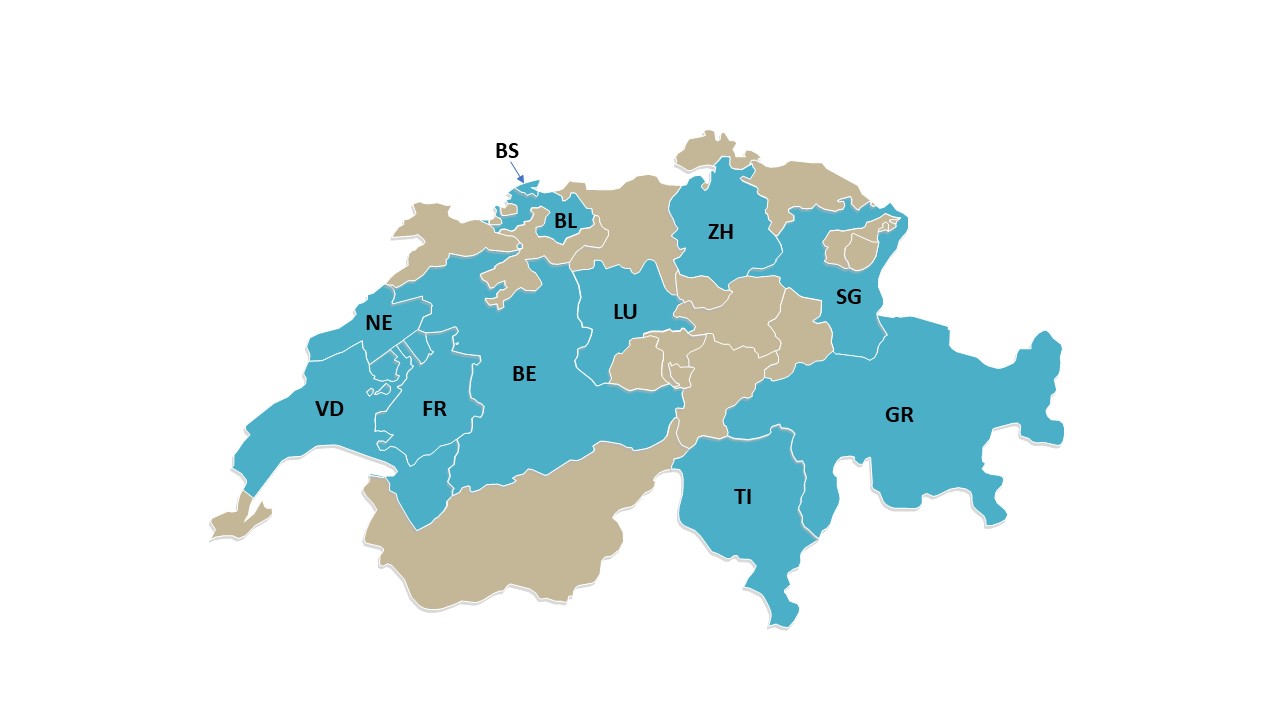
**

Note: Cantons in blue are the cantons included in this study

Abbreviations: BL= Basel**-**Landschaft; BS= Basel-Stadt; BE= Bern; FR= Fribourg; GR= Grisons; LU= Lucerne; NE= Neuchâtel; SG= Saint Gallen; TI= Ticino; VD= Vaud; ZU= Zürich

# **Figure S2. Flow chart of participants included in the analysis of seroprevalence trends (objective 1) and in the analysis for risk factors (objective 2), Corona Immunitas study, Switzerland, May 2020 - September 2021**

**
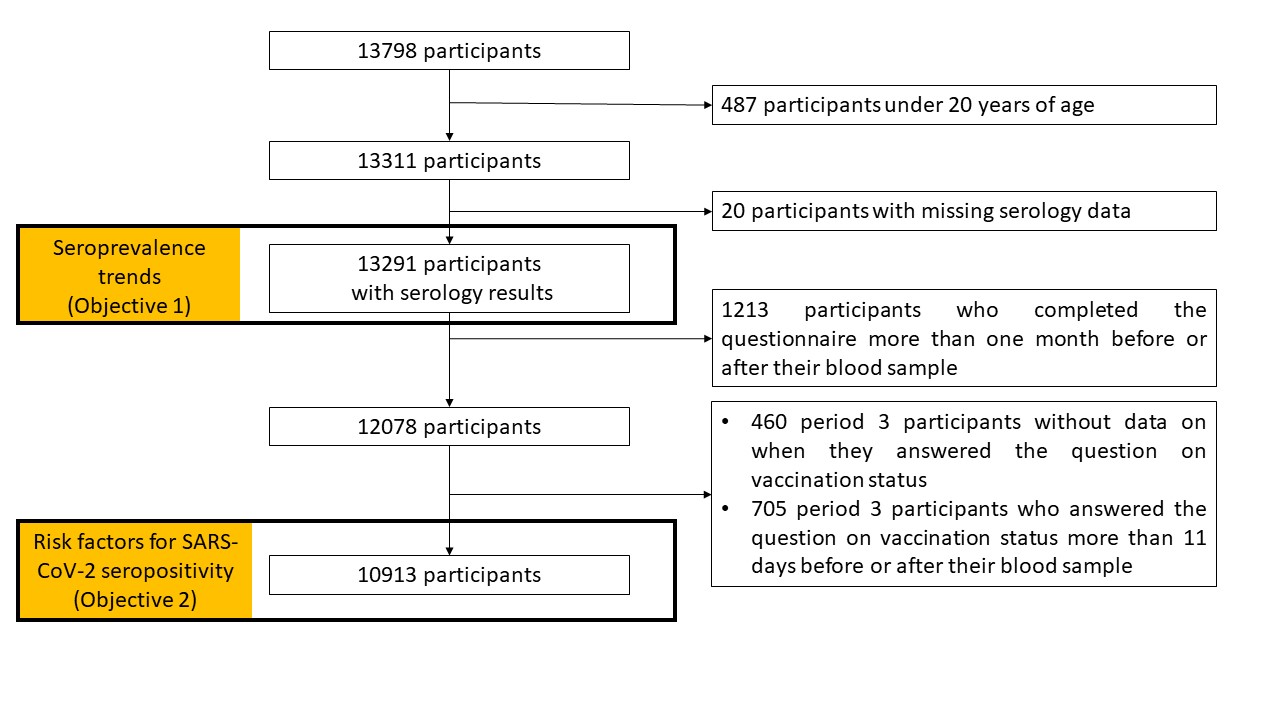
** Note: 65500 participants were invited to participate (response rate around 21%) **Table S1: Characteristics of participants across study periods, Corona Immunitas study, Switzerland, May 2020 – September 2021**

|  |  | **Overall** | **Period 1** | **Period 2** | **Period 3** |
| --- | --- | --- | --- | --- | --- |
| Time window | | 01/05/2020-31/09/2021 | 01/05/2020- 31/10/2020 | 01/11/2020- 15/05/2021 | 16/05/2021-31/09/2021 |
|  | | n (%) | n (%) | n (%) | n (%) |
|  | | 13291 (100) | 3402 (26) | 5611 (42) | 4278(32) |
| Canton | |  |  |  |  |
|  | Basel-Landschaft | 1187 (9) | 308 (26) | 577 (49) | 302 (25) |
|  | Basel-Stadt | 1255 (9) | 373 (30) | 515 (41) | 367 (29) |
|  | Bern | 1038 (8) | NA | 400 (39) | 638 (61) |
|  | Fribourg | 1473 (11) | 416 (28) | 551 (37) | 506 (34) |
|  | Grisons | 591 (4) | NA | 301 (51) | 290 (49) |
|  | Lucerne | 1045 (8) | NA | 438 (42) | 607 (58) |
|  | Neuchâtel | 1504 (11) | 382 (25) | 560 (37) | 562 (37) |
|  | Saint Gallen | 569 (4) | NA | 259 (46) | 310 (54) |
|  | Ticino | 1062 (18) | 643 (61) | 419 (39) | NA |
|  | Vaud | 1362 (10) | 412 (30) | 950 (70) | NA |
|  | Zürich | 2205 (17) | 868 (39) | 641 (29) | 696 (32) |
| **Sociodemographic characteristics** | | | | | |
| Sex [n=13279] | |  |  |  |  |
|  | Female | 6997 (53) | 1801 (53) | 2922 (52) | 2274 (53) |
|  | Male | 6282 (47) | 1597 (47) | 2686 (48) | 1999 (47) |
| Age group [n=13290] | | |  |  |  |
|  | ≥ 65 | 5240 (39) | 1097 (32) | 2450 (44) | 1693 (40) |
|  | 20-64 | 8050 (61) | 2305 (68) | 3160 (56) | 2585 (60) |
| Children in the household [n=13110] | | | |  |  |
|  | No children | 10050 (77) | 2488 (74) | 4335 (78) | 3227 (77) |
|  | One child | 1240 (9) | 392 (12) | 503 (9) | 345 (8) |
|  | More than one child | 1820 (14) | 488 (14) | 711 (13) | 621 (15) |
| **Socioeconomic characteristics** | | | | | |
| Educational level^a^ [n=12816] | | |  |  |  |
|  | Tertiary | 5855 (46) | 1419 (47) | 2548 (46) | 1888 (45) |
|  | Secondary | 6193 (48) | 1460 (48) | 2656 (48) | 2077 (49) |
|  | Primary | 768 (6) | 152 (5) | 359 (6) | 257 (6) |
| Household income [n=11821] | | |  |  |  |
|  | > CHF 9'000 | 4072 (34) | 1000 (37) | 1775 (34) | 1297 (33) |
|  | CHF>6'000- 9'000 | 3261 (28) | 764 (28) | 1460 (28) | 1037 (26) |
|  | CHF>3'000- 6'000 | 3326 (28) | 722 (27) | 1413 (27) | 1191 (30) |
|  | CHF≤ 3'000 | 1162 (10) | 229 (8) | 499 (10) | 434 (11) |
| Employment status [n=12944] | | |  |  |  |
|  | Retired | 4875 (37) | 1134 (35) | 2040 (37) | 1701 (41) |
|  | Outside the labour force^b^ | 1265 (10) | 269 (8) | 771 (14) | 225 (5) |
|  | Self employed | 1320 (10) | 623 (19) | 394 (7) | 303 (7) |
|  | Employed | 5484 (42) | 1248 (38) | 2295 (42) | 1941 (47) |
| **Health status characteristics** | | | | | |
| Body Mass Index [n=13059] | | |  |  |  |
|  | <18.5 | 347 (3) | 90 (3) | 140 (3) | 117 (3) |
|  | 18.5 – 24.9 | 6837 (52) | 1768 (53) | 2896 (52) | 2173 (52) |
|  | 25 – 29.9 | 4308 (33) | 1069 (32) | 1836 (33) | 1403 (34) |
|  | ≥ 30 | 1567 (12) | 423 (13) | 654 (12) | 490 (12) |
| Comorbiditiy score ^c^ [n=12722] | | |  |  |  |
|  | 0 | 6730 (53) | 1597 (54) | 2883 (52) | 2250 (53) |
|  | 1 | 4054 (32) | 923 (31) | 1785 (32) | 1346 (32) |
|  | ≥2 | 1938 (15) | 448 (15) | 876 (16) | 614 (15) |
| Smoking [n=12790] | | |  |  |  |
|  | Non-smoker | 10789 (84) | 2448 (82) | 4739 (85) | 3602 (85) |
|  | Smoker | 2001 (16) | 528 (18) | 843 (15) | 630 (15) |
| **Preventive behaviours** | | | | | |
| Physical distancing during previous 7 days [n=12096] | | | | |  |
|  | Frequently | 11034 (91) | 2496 (91) | 4914 (95) | 3624 (86) |
|  | Occasionally | 687 (6) | 162 (6) | 147 (3) | 378 (9) |
|  | Rarely | 375 (3) | 73 (3) | 86 (2) | 216 (5) |
| Staying at home during previous 7 days [n=11703] | | | |  |  |
|  | Frequently | 8051 (69) | 1290 (55) | 4188 (81) | 2573 (61) |
|  | Occasionally | 1950 (17) | 475 (20) | 599 (12) | 876 (21) |
|  | Rarely | 1702 (14) | 572 (24) | 360 (7) | 770 (18) |
| Wearing mask during previous 7 days [n=11929] | | | |  |  |
|  | Frequently | 9898 (83) | 1237 (48) | 4900 (95) | 3761 (89) |
|  | Occasionally | 1240 (10) | 668 (26) | 208 (4) | 364 (9) |
|  | Rarely | 791 (7) | 664 (26) | 34 (1) | 93 (2) |
| Hygiene measures during previous 7 days [n=12108] | | | | |  |
|  | Frequently | 11355 (94) | 2643 (97) | 4947 (96) | 3765 (89) |
|  | Occasionally/rarely | 752 (6) | 93 (4) | 202 (4) | 458 (11) |
| ^a^ International Standard Classification of Education (ISCED)  ^b^ Outside the labour force includes participants in training/studying and not employed participants  ^c^ Comorbidity score goes from 0 to ≥2 and was calculated using the following possible answers: cancer; immunological diseases; cardiovascular diseases or diabetes or hypertension; respiratory diseases; allergies | | | | | |

**Table S2: Characteristics of participants by canton, Corona Immunitas study, Switzerland, May 2020 – September 2021**

Click on the links below to go directly to the desired canton:

[Basel-Stadt](#Baselstadt) [Bern](#Bern) [Fribourg](#Fribourg) [Grisons](#Grisons) [Lucerne](#Lucerne) [Neuchâtel](#Neuchatel) [Saint Gallen](#SG) [Ticino](#Ticino) [Vaud](#Vaud) [Zürich](#Zurich)

| **Basel-Landschaft** | | n (%) |
| --- | --- | --- |
| Sex |  |  |
|  | Female | 633 (53) |
|  | Male | 554 (47) |
| Age |  |  |
|  | ≥ 65 | 353(30) |
|  | 20-64 | 834(70) |
| Children in the household | |  |
|  | No children | 894(76) |
|  | One child | 121(10) |
|  | More than one child | 165(14) |
| Educational level | |  |
|  | Tertiary | 552(47) |
|  | Secondary | 583(49) |
|  | Primary | 49(4) |
| Household income | |  |
|  | > CHF 9'000 | 421(37) |
|  | CHF>6'000- 9'000 | 333(30) |
|  | CHF>3'000- 6'000 | 275(24) |
|  | CHF≤ 3'000 | 100(9) |
| Employment status | |  |
|  | Retired | 386(33) |
|  | Outside the labour force | 111(9) |
|  | Self employed | 82(7) |
|  | Employed | 598(51) |
| Body Mass Index | |  |
|  | <18.5 | 30(3) |
|  | 18.5 – 24.9 | 602(51) |
|  | 25-29.9 | 375(32) |
|  | ≥ 30 | 171(15) |
| Comorbidity score | |  |
|  | 0 | 608(51) |
|  | 1 | 394(33) |
|  | ≥2 | 183(15) |
| Smoking | |  |
|  | Non-smoker | 1026(87) |
|  | Smoker | 160(13) |
| Physical distancing during previous 7 days | |  |
|  | Frequently | 1089(92) |
|  | Occasionally/rarely | 94(8) |
| Staying at home during previous 7 days | |  |
|  | Frequently | 830(70) |
|  | Occasionally/rarely | 354(30) |
| Wearing mask during previous 7 days | |  |
|  | Frequently | 1028(87) |
|  | Occasionally/rarely | 151(13) |
| Hygiene measures during previous 7 days | |  |
|  | Frequently | 1131(96) |
|  | Occasionally/rarely | 53(5) |
| **Basel-Stadt** | | |
| Sex |  |  |
|  | Female | 720(57) |
|  | Male | 534(43) |
| Age |  |  |
|  | ≥ 65 | 332(26) |
|  | 20-64 | 923(74) |
| Children in the household | |  |
|  | No children | 986(79) |
|  | One child | 104(8) |
|  | More than one child | 155(12) |
| Educational level | |  |
|  | Tertiary | 809(65) |
|  | Secondary | 407(33) |
|  | Primary | 35(3) |
| Household income | |  |
|  | > CHF 9'000 | 479(40) |
|  | CHF>6'000- 9'000 | 301(25) |
|  | CHF>3'000- 6'000 | 305(25) |
|  | CHF≤ 3'000 | 119(10) |
| Employment status | |  |
|  | Retired | 354(28) |
|  | Outside the labour force | 107(9) |
|  | Self employed | 126(10) |
|  | Employed | 660(53) |
| Body Mass Index | |  |
|  | <18.5 | 29(2) |
|  | 18.5 – 24.9 | 721(58) |
|  | 25-29.9 | 376(30) |
|  | ≥ 30 | 120(10) |
| Comorbidity score | |  |
|  | 0 | 636(51) |
|  | 1 | 431(34) |
|  | ≥2 | 182(15) |
| Smoking | |  |
|  | Non-smoker | 1019(81) |
|  | Smoker | 234(19) |
| Physical distancing during previous 7 days | |  |
|  | Frequently | 1147(92) |
|  | Occasionally/rarely | 102(8) |
| Staying at home during previous 7 days | |  |
|  | Frequently | 791(63) |
|  | Occasionally/rarely | 458(37) |
| Wearing mask during previous 7 days | |  |
|  | Frequently | 1112(89) |
|  | Occasionally/rarely | 136(11) |
| Hygiene measures during previous 7 days | |  |
|  | Frequently | 1190(96) |
|  | Occasionally/rarely | 56(5) |
| **Bern** | | |
| Sex |  |  |
|  | Female | 506(50) |
|  | Male | 528(50) |
| Age |  |  |
|  | ≥ 65 | 497(48) |
|  | 20-64 | 541(52) |
| Children in the household | |  |
|  | No children | 795(80) |
|  | One child | 69(7) |
|  | More than one child | 125(13) |
| Educational level | |  |
|  | Tertiary | 401(40) |
|  | Secondary | 542(54) |
|  | Primary | 56(6) |
| Household income | |  |
|  | > CHF 9'000 | 247(27) |
|  | CHF>6'000- 9'000 | 262(28) |
|  | CHF>3'000- 6'000 | 288(31) |
|  | CHF≤ 3'000 | 126(14) |
| Employment status | |  |
|  | Retired | 465(47) |
|  | Outside the labour force | 38(4) |
|  | Self employed | 72(7) |
|  | Employed | 411(42) |
| Body Mass Index | |  |
|  | <18.5 | 25(3) |
|  | 18.5 – 24.9 | 500(51) |
|  | 25-29.9 | 325(33) |
|  | ≥ 30 | 131(13) |
| Comorbidity score | |  |
|  | 0 | 528(53) |
|  | 1 | 297(30) |
|  | ≥2 | 163(17) |
| Smoking | |  |
|  | Non-smoker | 856(85) |
|  | Smoker | 149(15) |
| Physical distancing during previous 7 days | |  |
|  | Frequently | 889(89) |
|  | Occasionally/rarely | 109(11) |
| Staying at home during previous 7 days | |  |
|  | Frequently | 651(65) |
|  | Occasionally/rarely | 352(35) |
| Wearing mask during previous 7 days | |  |
|  | Frequently | 903(90) |
|  | Occasionally/rarely | 97(10) |
| Hygiene measures during previous 7 days | |  |
|  | Frequently | 925(92) |
|  | Occasionally/rarely | 78(8) |
| **Fribourg** | | |
| Sex |  |  |
|  | Female | 799(54) |
|  | Male | 673(46) |
| Age |  |  |
|  | ≥ 65 | 673(46) |
|  | 20-64 | 800(54) |
| Children in the household | |  |
|  | No children | 1071(74) |
|  | One child | 142(10) |
|  | More than one child | 239(16) |
| Educational level | |  |
|  | Tertiary | 615(42) |
|  | Secondary | 739(50) |
|  | Primary | 115(8) |
| Household income | |  |
|  | > CHF 9'000 | 528(37) |
|  | CHF>6'000- 9'000 | 400(28) |
|  | CHF>3'000- 6'000 | 388(27) |
|  | CHF≤ 3'000 | 98(7) |
| Employment status | |  |
|  | Retired | 694(48) |
|  | Outside the labour force | 72(5) |
|  | Self employed | 82(6) |
|  | Employed | 605(42) |
| Body Mass Index | |  |
|  | <18.5 | 42(3) |
|  | 18.5 – 24.9 | 742(51) |
|  | 25-29.9 | 476(32) |
|  | ≥ 30 | 205(14) |
| Comorbidity score | |  |
|  | 0 | 781(54) |
|  | 1 | 442(30) |
|  | ≥2 | 234(16) |
| Smoking | |  |
|  | Non-smoker | 1270(86) |
|  | Smoker | 200(14) |
| Physical distancing during previous 7 days | |  |
|  | Frequently | 1348(92) |
|  | Occasionally/rarely | 119(8) |
| Staying at home during previous 7 days | |  |
|  | Frequently | 1034(71) |
|  | Occasionally/rarely | 430(29) |
| Wearing mask during previous 7 days | |  |
|  | Frequently | 1122(77) |
|  | Occasionally/rarely | 342(23) |
| Hygiene measures during previous 7 days | |  |
|  | Frequently | 1314(90) |
|  | Occasionally/rarely | 148(10) |
| **Grisons** | | |
| Sex |  |  |
|  | Female | 319(54) |
|  | Male | 271(46) |
| Age |  |  |
|  | ≥ 65 | NA |
|  | 20-64 | 591(100) |
| Children in the household | |  |
|  | No children | 373(64) |
|  | One child | 76(13) |
|  | More than one child | 136(23) |
| Educational level | |  |
|  | Tertiary | 244(42) |
|  | Secondary | 306(52) |
|  | Primary | 37(6) |
| Household income | |  |
|  | > CHF 9'000 | 157(29) |
|  | CHF>6'000- 9'000 | 146(27) |
|  | CHF>3'000- 6'000 | 167(31) |
|  | CHF≤ 3'000 | 70(13) |
| Employment status | |  |
|  | Retired | 14(2) |
|  | Outside the labour force | 46(8) |
|  | Self employed | 77(13) |
|  | Employed | 438(76) |
| Body Mass Index | |  |
|  | <18.5 | 14(2) |
|  | 18.5 – 24.9 | 331(57) |
|  | 25-29.9 | 187(32) |
|  | ≥ 30 | 50(9) |
| Comorbidity score | |  |
|  | 0 | 391(67) |
|  | 1 | 159(27) |
|  | ≥2 | 38(6) |
| Smoking | |  |
|  | Non-smoker | 484(82) |
|  | Smoker | 104(18) |
| Physical distancing during previous 7 days | |  |
|  | Frequently | 519(88) |
|  | Occasionally/rarely | 68(12) |
| Staying at home during previous 7 days | |  |
|  | Frequently | 400(68) |
|  | Occasionally/rarely | 185(32) |
| Wearing mask during previous 7 days | |  |
|  | Frequently | 526(90) |
|  | Occasionally/rarely | 59(10) |
| Hygiene measures during previous 7 days | |  |
|  | Frequently | 535(92) |
|  | Occasionally/rarely | 49(8) |
| **Lucerne** | | |
| Sex |  |  |
|  | Female | 514(49) |
|  | Male | 530(51) |
| Age |  |  |
|  | ≥ 65 | 507(49) |
|  | 20-64 | 537(51) |
| Children in the household | |  |
|  | No children | 798(79) |
|  | One child | 81(8) |
|  | More than one child | 136(13) |
| Educational level | |  |
|  | Tertiary | 407(40) |
|  | Secondary | 554(54) |
|  | Primary | 63(6) |
| Household income | |  |
|  | > CHF 9'000 | 254(27) |
|  | CHF>6'000- 9'000 | 270(28) |
|  | CHF>3'000- 6'000 | 301(32) |
|  | CHF≤ 3'000 | 129(14) |
| Employment status | |  |
|  | Retired | 495(48) |
|  | Outside the labour force | 45(4) |
|  | Self employed | 70(7) |
|  | Employed | 411(40) |
| Body Mass Index | |  |
|  | <18.5 | 29(3) |
|  | 18.5 – 24.9 | 503(50) |
|  | 25-29.9 | 358(35) |
|  | ≥ 30 | 125(12) |
| Comorbidity score | |  |
|  | 0 | 547(53) |
|  | 1 | 307(30) |
|  | ≥2 | 169(17) |
| Smoking | |  |
|  | Non-smoker | 891(86) |
|  | Smoker | 139(14) |
| Physical distancing during previous 7 days | |  |
|  | Frequently | 927(91) |
|  | Occasionally/rarely | 92(9) |
| Staying at home during previous 7 days | |  |
|  | Frequently | 818(80) |
|  | Occasionally/rarely | 206(20) |
| Wearing mask during previous 7 days | |  |
|  | Frequently | 960(94) |
|  | Occasionally/rarely | 61(6) |
| Hygiene measures during previous 7 days | |  |
|  | Frequently | 955(93) |
|  | Occasionally/rarely | 71(7) |
| **Neuchâtel** | | |
| Sex |  |  |
|  | Female | 797(53) |
|  | Male | 707(47) |
| Age |  |  |
|  | ≥ 65 | 718(48) |
|  | 20-64 | 786(52) |
| Children in the household | |  |
|  | No children | 1160(77) |
|  | One child | 140(9) |
|  | More than one child | 197(13) |
| Educational level | |  |
|  | Tertiary | 655(44) |
|  | Secondary | 720(48) |
|  | Primary | 122(8) |
| Household income | |  |
|  | > CHF 9'000 | 486(34) |
|  | CHF>6'000- 9'000 | 382(27) |
|  | CHF>3'000- 6'000 | 442(31) |
|  | CHF≤ 3'000 | 117(8) |
| Employment status | |  |
|  | Retired | 740(51) |
|  | Outside the labour force | 84(6) |
|  | Self employed | 86(6) |
|  | Employed | 549(38) |
| Body Mass Index | |  |
|  | <18.5 | 41(3) |
|  | 18.5 – 24.9 | 730(49) |
|  | 25-29.9 | 508(34) |
|  | ≥ 30 | 206(14) |
| Comorbidity score | |  |
|  | 0 | 789(53) |
|  | 1 | 466(31) |
|  | ≥2 | 243(16) |
| Smoking | |  |
|  | Non-smoker | 1269(85) |
|  | Smoker | 230(15) |
| Physical distancing during previous 7 days | |  |
|  | Frequently | 1355(90) |
|  | Occasionally/rarely | 144(10) |
| Staying at home during previous 7 days | |  |
|  | Frequently | 927(62) |
|  | Occasionally/rarely | 572(38) |
| Wearing mask during previous 7 days | |  |
|  | Frequently | 1227(82) |
|  | Occasionally/rarely | 271(18) |
| Hygiene measures during previous 7 days | |  |
|  | Frequently | 1437(96) |
|  | Occasionally/rarely | 61(4) |
| **Saint Gallen** | | |
| Sex |  |  |
|  | Female | 312 (55) |
|  | Male | 255(45) |
| Age |  |  |
|  | ≥ 65 | NA |
|  | 20-64 | 569(100) |
| Children in the household | |  |
|  | No children | 362(64) |
|  | One child | 75(13) |
|  | More than one child | 128(23) |
| Educational level | |  |
|  | Tertiary | 258(46) |
|  | Secondary | 280(50) |
|  | Primary | 25(4) |
| Household income | |  |
|  | > CHF 9'000 | 170(33) |
|  | CHF>6'000- 9'000 | 143(28) |
|  | CHF>3'000- 6'000 | 150(29) |
|  | CHF≤ 3'000 | 48(9) |
| Employment status | |  |
|  | Retired | 15(3) |
|  | Outside the labour force | 54(10) |
|  | Self employed | 9(48) |
|  | Employed | 438(79) |
| Body Mass Index | |  |
|  | <18.5 | 14(2) |
|  | 18.5 – 24.9 | 308(55) |
|  | 25-29.9 | 191(34) |
|  | ≥ 30 | 50(9) |
| Comorbidity score | |  |
|  | 0 | 362(64) |
|  | 1 | 165(29) |
|  | ≥2 | 39(7) |
| Smoking | |  |
|  | Non-smoker | 460(81) |
|  | Smoker | 106(19) |
| Physical distancing during previous 7 days | |  |
|  | Frequently | 491(87) |
|  | Occasionally/rarely | 73(13) |
| Staying at home during previous 7 days | |  |
|  | Frequently | 351(62) |
|  | Occasionally/rarely | 212(38) |
| Wearing mask during previous 7 days | |  |
|  | Frequently | 492(87) |
|  | Occasionally/rarely | 74(13) |
| Hygiene measures during previous 7 days | |  |
|  | Frequently | 508(90) |
|  | Occasionally/rarely | 58(10) |
| **Ticino** | | |
| Sex |  |  |
|  | Female | 592(56) |
|  | Male | 468(44) |
| Age |  |  |
|  | ≥ 65 | 421(40) |
|  | 20-64 | 641(60) |
| Children in the household | |  |
|  | No children | 818(78) |
|  | One child | 110(11) |
|  | More than one child | 118(11) |
| Educational level | |  |
|  | Tertiary | 361(34) |
|  | Secondary | 613(58) |
|  | Primary | 74(7) |
| Household income | |  |
|  | > CHF 9'000 | 274(33) |
|  | CHF>6'000- 9'000 | 252(30) |
|  | CHF>3'000- 6'000 | 253(30) |
|  | CHF≤ 3'000 | 62(7) |
| Employment status | |  |
|  | Retired | 43(4) |
|  | Outside the labour force | 491(50) |
|  | Self employed | 426(43) |
|  | Employed | 27(3) |
| Body Mass Index | |  |
|  | <18.5 | 29(3) |
|  | 18.5 – 24.9 | 548(52) |
|  | 25-29.9 | 357(34) |
|  | ≥ 30 | 112(11) |
| Comorbidity score | |  |
|  | 0 | 578(56) |
|  | 1 | 339(33) |
|  | ≥2 | 121(12) |
| Smoking | |  |
|  | Non-smoker | 858(82) |
|  | Smoker | 192(18) |
| Physical distancing during previous 7 days | |  |
|  | Frequently | NA |
|  | Occasionally/rarely | NA |
| Staying at home during previous 7 days | |  |
|  | Frequently | NA |
|  | Occasionally/rarely | NA |
| Wearing mask during previous 7 days | |  |
|  | Frequently | NA |
|  | Occasionally/rarely | NA |
| Hygiene measures during previous 7 days | |  |
|  | Frequently | NA |
|  | Occasionally/rarely | NA |
| **Vaud** | | |
| Sex |  |  |
|  | Female | 704(52) |
|  | Male | 658(48) |
| Age |  |  |
|  | ≥ 65 | 599(44) |
|  | 20-64 | 763(56) |
| Children in the household | |  |
|  | No children | 1009(74) |
|  | One child | 152(11) |
|  | More than one child | 196(14) |
| Educational level | |  |
|  | Tertiary | 493(49) |
|  | Secondary | 439(43) |
|  | Primary | 84(8) |
| Household income | |  |
|  | > CHF 9'000 | 324(38) |
|  | CHF>6'000- 9'000 | 255(28) |
|  | CHF>3'000- 6'000 | 199(23) |
|  | CHF≤ 3'000 | 78(9) |
| Employment status | |  |
|  | Retired | 587(44) |
|  | Outside the labour force | 114(9) |
|  | Self employed | 90(7) |
|  | Employed | 531(40) |
| Body Mass Index | |  |
|  | <18.5 | 42(3) |
|  | 18.5 – 24.9 | 696(52) |
|  | 25-29.9 | 442(33) |
|  | ≥ 30 | 169(13) |
| Comorbidity score | |  |
|  | 0 | 491(52) |
|  | 1 | 288(30) |
|  | ≥2 | 169(18) |
| Smoking | |  |
|  | Non-smoker | 796(84) |
|  | Smoker | 153(16) |
| Physical distancing during previous 7 days | |  |
|  | Frequently | 1292(97) |
|  | Occasionally/rarely | 45(3) |
| Staying at home during previous 7 days | |  |
|  | Frequently | 757(80) |
|  | Occasionally/rarely | 186(20) |
| Wearing mask during previous 7 days | |  |
|  | Frequently | 929(79) |
|  | Occasionally/rarely | 251(21) |
| Hygiene measures during previous 7 days | |  |
|  | Frequently | 1324(98) |
|  | Occasionally/rarely | 28(2) |
| **Zurich** | | |
| Sex |  |  |
|  | Female | 1101(50) |
|  | Male | 1104(50) |
| Age |  |  |
|  | ≥ 65 | 1138(52) |
|  | 20-64 | 1067(48) |
| Children in the household | |  |
|  | No children | 1784(82) |
|  | One child | 170(8) |
|  | More than one child | 225(10) |
| Educational level | |  |
|  | Tertiary | 1060(49) |
|  | Secondary | 1010(46) |
|  | Primary | 108(5) |
| Household income | |  |
|  | > CHF 9'000 | 732(36) |
|  | CHF>6'000- 9'000 | 517(26) |
|  | CHF>3'000- 6'000 | 558(28) |
|  | CHF≤ 3'000 | 215(11) |
| Employment status | |  |
|  | Retired | 1082(50) |
|  | Outside the labour force | 103(5) |
|  | Self employed | 161(7) |
|  | Employed | 816(38) |
| Body Mass Index | |  |
|  | <18.5 | 50(2) |
|  | 18.5 – 24.9 | 1157(54) |
|  | 25-29.9 | 713(33) |
|  | ≥ 30 | 229(11) |
| Comorbidity score | |  |
|  | 0 | 1019(47) |
|  | 1 | 766(35) |
|  | ≥2 | 397(18) |
| Smoking | |  |
|  | Non-smoker | 1860(85) |
|  | Smoker | 334(15) |
| Physical distancing during previous 7 days | |  |
|  | Frequently | 1977(90) |
|  | Occasionally/rarely | 216(10) |
| Staying at home during previous 7 days | |  |
|  | Frequently | 1492(68) |
|  | Occasionally/rarely | 697(32) |
| Wearing mask during previous 7 days | |  |
|  | Frequently | 1599(73) |
|  | Occasionally/rarely | 589(27) |
| Hygiene measures during previous 7 days | |  |
|  | Frequently | 2036(93) |
|  | Occasionally/rarely | 151(7) |

Note: NA=not available; preventive behaviors data from Ticino and data from Vaud period 1 were not reported because not harmonizable with data from other sites

**Table S3: Weighted seroprevalence (IgG antispike) by study period, stratified by sex and age, Corona Immunitas study, Switzerland, May 2020 – September 2021**

|  | **Period 1**  (01/05/2020- 31/10/2020) | **Period 2**  (01/11/2020- 15/05/2021) | **Period 3**  (16/05/2021-31/09/2021) |
| --- | --- | --- | --- |
|  | % (95%CI) | % (95%CI) | % (95%CI) |
| **National level** |  |  |  |
| 20-64 | 4.1(2.5-5.5) | 15.8(13.9-17.4) | 65.9(63.8-68.1) |
| +65 | 2.3(0.7-3.9) | 17.1(15.0-19.0) | 92.0(90.2-94.2) |
| F | 3.7(2.1-5.1) | 15.6(13.6-17.3) | 71.6(69.3-73.9) |
| M | 3.8(1.9-5.3) | 16.7(14.6-18.5) | 72.5(70.1-74.9) |
| **Basel-Landschaft** |  |  |  |
| 20-64 | 3.1(1.3-5.8) | 12.8(9.5-16.6) | 79.4(73.2-84.9) |
| +65 | 2.2(0.4-7.2) | 26.3(19.8-33.3) | 92.0(85.7-96.5) |
| F | 3.6(1.4-7.3) | 14.8(10.9-19.2) | 80.0(73.3-85.7) |
| M | 2.0 (0.5-5.1) | 18.3(13.8-23.4) | 85.9(78.8-91.8) |
| **Basel-Stadt** |  |  |  |
| 20-64 | 4.7(2.4-7.8) | 14.0(10.4-17.9) | 71.6(65.9-77.2) |
| +65 | 5.4(1.6-11.7) | 37.8(30.0-45.8) | 95.2(89.9-98.2) |
| F | 5.4(2.7-9.0) | 20.1(15.8-24.7) | 77.8(71.8-83.2) |
| M | 4.3(1.5-8.4) | 19.2(14.2-24.7) | 76.6(69.8-82.9) |
| **Bern** |  |  |  |
| 20-64 | NA | 11.1(6.9-15.8) | 74.5(69.4-79.4) |
| +65 | NA | 10.2(5.9-15.4) | 88.7(84.3-92.5) |
| F | NA | 12.2(7.9-17.8) | 79.6(74.5-84.4) |
| M | NA | 9.1(5.2-14.1) | 76.8(71.0-82.0) |
| **Fribourg** |  |  |  |
| 20-64 | 6.5(3.5-10.5) | 19.4(15.0-24.2) | 69.2(62.9-75.0) |
| +65 | 3.0(0.9-6.4) | 34.5(28.5-40.7) | 90.5(85.8-94.2) |
| F | 5.5(2.8-9.4) | 21.8(17.1-26.8) | 71.6(65.7-77.3) |
| M | 6.0(2.5-11.1) | 23.2(18.1-29.1) | 75.4(67.7-82.5) |
| **Grisons ^a^** |  |  |  |
| F | NA | 13.4(8.5-19.3) | 43.0(35.2-51.0) |
| M | NA | 17.8(11.9-24.8) | 43.4(34.8-52.3) |
| **Lucerne** |  |  |  |
| 20-64 | NA | 15.9(11.3-21.0) | 49.6(43.9-55.6) |
| +65 | NA | 14.0(9.2-19.8) | 91.0(87.2-94.4) |
| F | NA | 16.8(11.6-22.5) | 58.5(53.0-64.4) |
| M | NA | 14.1(9.3-19.6) | 59.2(52.4-66.3) |
| **Neuchâtel** |  |  |  |
| 20-64 | 3.3(1.4-6.1) | 19.7(15.3-24.8) | 74.8(69.3-80.0) |
| +65 | 2.0(0.4-5.1) | 17.2(12.6-22.2) | 92.9(89.0-96.2) |
| F | 4.1(1.8-7.5) | 17.8(13.3-22.9) | 76.6(71.0-81.9) |
| M | 1.7(0.4-4.6) | 20.4(15.3-26.1) | 82.1(75.4-87.6) |
| **Saint Gallen ^a^** |  |  |  |
| F | NA | 12.8(7.6-19.2) | 62.2(54.6-69.5) |
| M | NA | 10.4(5.5-17.0) | 62.5(54.-70.3) |
| **Ticino ^b^** |  |  |  |
| F | 7.9(5.2-11.3) | 7.6(4.3-11.6) | NA |
| M | 7.4(4.2-11.2) | 5.8(2.6-10.0) | NA |
| **Vaud** |  |  |  |
| 20-64 | 6.7(4.1-9.7) | 25.2(21.3-29.3) | NA |
| +65 | 6.8(4.2-9.8) | 17.9(13.9-22.0) | NA |
| F | 7.6(4.3-11.6) | 23.5(19.5-27.8) | NA |
| M | 5.8(2.6-10.0) | 23.7(19.3-28.5) | NA |
| **Zurich** |  |  |  |
| 20-64 | 2.2(1.0-3.9) | 9.5(6.1-13.7) | 74.7(69.4-79.5)) |
| +65 | 1.7(0.4-3.6) | 10.0(6.8-13.5) | 92.2(89.3-95.8) |
| F | 2.2(1.0-4.0) | 8.9(5.7-12.9) | 76.7(71.4-81.8) |
| M | 2.0(0.5-4.1) | 10.4(6.6-15.0) | 80.4(74.7-85.7) |

Note: F= Females, M= Males

^a^ In cantons Grisons and Saint Gallen, only participants aged from 20 to 64 years were tested

^b^ In canton Ticino, during period 1, only participants aged from 20 to 64 were tested. During period 2 only people aged 65 years and over were tested

**Table S4: Missing data for sociodemographic, socioeconomic and health status variables by study period (objective 2), Corona Immunitas study, Switzerland, May 2020 – September 2021**

|  | **Period 1 n=3108** | **Period 2 n=4969** | **Period 3 n=2836** |
| --- | --- | --- | --- |
| **Sociodemographic characteristics** | n (%) | n (%) | n (%) |
| Sex | 4 (0) | 3 (0) | 3 (0) |
| Age group | 0 (0) | 1 (0) | 0 (0) |
| Children in the household | 29 (1) | 50 (1) | 20 (1) |
| **Socioeconomic characteristics** | |  |  |
| Educational level | 294 (9) | 40 (1) | 7 (0) |
| Household Income | 575 (19) | 396 (8) | 169 (6) |
| Employment status | 119 (4) | 91 (2) | 45 (2) |
| **Health status** | |  |  |
| Body Mass Index | 47 (2) | 78 (2) | 35 (1) |
| Comorbiditiy score | 335 (11) | 53 (1) | 2 (0) |
| Smoking | 328 (11) | 25 (1) | 0 (0) |

#

# **Table S5: Missing data for preventive behaviours variables by study period (objective 2), Corona Immunitas study, Switzerland, May 2020 – September 2021**

|  | **Period 1 n=2151** | **Period 2 n=4969** | **Period 3 n=2836** |
| --- | --- | --- | --- |
| **Preventive behaviours** | n (%) | n (%) | n (%) |
| Physical distancing during previous 7 days | 6 (0) | 267 (5) | 5 (0) |
| Staying at home during previous 7 days | 9 (0) | 267 (5) | 6 (0) |
| Wearing mask during previous 7 days | 10 (0) | 270 (5) | 8 (0) |
| Hygiene measures during previous 7 days | 14 (1) | 264 (5) | 6 (0) |

**Table S6: percentages of vaccinated participants (period 3, objective 2), Corona Immunitas study, Switzerland**

|  | | | **N** | **Vaccinated** |
| --- | --- | --- | --- | --- |
| **Sociodemographic factors** | | |  |  |
| Sex | | | 2826 | 69% |
|  | Female | |  | 68% |
|  | Male | |  | 71% |
| Age groups | | | 2829 | 69% |
|  | ≥ 65 | |  | 91% |
|  | 20-64 | |  | 59% |
| Children in the household | | | 2809 | 69% |
|  | No children | |  | 74% |
|  | One child | |  | 58% |
|  | More than one child | |  | 52% |
| **Socioeconomic characteristics** | | |  |  |
| Educational level | | | 2822 | 69% |
|  | Tertiary | |  | 74% |
|  | Secondary | |  | 66% |
|  | Primary | |  | 61% |
| Income | | | 2663 | 69% |
|  | > CHF 9'000 | |  | 77% |
|  | CHF>6'000- 9'000 | |  | 68% |
|  | CHF>3'000- 6'000 | |  | 65% |
|  | CHF≤ 3'000 | |  | 57% |
| Employment status | | | 2784 | 69% |
|  | Retired | |  | 91% |
|  | Outside the labour force^b^ | |  | 54% |
|  | Self employed | |  | 55% |
|  | Employed | |  | 60% |
| **Health status** | | |  |  |
| Body Mass Index | | | 2795 | 69% |
|  | 18.5 – 24.9 | |  | 63% |
|  | <18.5 | |  | 67% |
|  | 25-29.9 | |  | 72% |
|  | ≥ 30 | |  | 74% |
| Comorbiditiy score^c^ | | | 2827 | 69% |
|  | 0 | |  | 63% |
|  | 1 | |  | 73% |
|  | ≥2 | |  | 86% |
| Smoking | | | 2829 | 69% |
|  | Non-smoker | |  | 71% |
|  | Smoker | |  | 59% |
| **Preventive behaviors** | | |  |  |
| Physical distancing during previous 7 days | | | 2824 | 69% |
|  | | Frequently |  | 73% |
|  | | Occasionally |  | 52% |
|  | | Rarely |  | 47% |
| Staying at home during previous 7 days^a^ | | | 2823 | 69% |
|  | | Frequently |  | 75% |
|  | | Occasionally |  | 64% |
|  | | Rarely |  | 58% |
| Wearing mask during previous 7 days | | | 2821 | 69% |
|  | | Frequently |  | 72% |
|  | | Occasionally |  | 51% |
|  | | Rarely |  | 31% |
| Hygiene measures during previous 7 days | | | 2823 | 69% |
|  | | Frequently |  | 72% |
|  | | Occasionally |  | 45% |
|  | | Rarely |  | 52% |

Note: for this table, the third answer option to the vaccination status question (“I don’t want to answer”) was excluded

^a^ International Standard Classification of Education (ISCED)

^b^ Outside the labour force includes participants in training/studying and not employed participants

^c^ Comorbidity score goes from 0 to ≥2 and was calculated using the following possible answers: cancer; immunological diseases; cardiovascular diseases or diabetes or hypertension; respiratory diseases; allergies

**Sensitivity analysis**

Sensitivity analyses including participants who had completed the baseline questionnaire more than 60 days before and after their blood sampling gave similar results as the main analyses (Tables S7-S8). Including a third age category (20-34 years; Table S9), we found a higher prevalence of seropositivity in people aged 20-34 compared to older people (PR= 2.87, 95% CI 1.23-6.71) during period 1 and a lower prevalence of seropositivity during period 3 (PR= 0.64, 95% CI 0.57-0.63). This last difference tended however to disappear after adjustment for vaccination status. We re-ran the models using a preventive behaviour score (Table S10). The lower the adherence to preventive behaviours, the lower the prevalence of seropositivity in period 3 (PR in people who rarely/occasionally adhered to all preventive behaviours VS people who frequently adhered to all of them = 0.64, 95% CI 0.50-0.81) . These differences disappeared upon adjustment for vaccination status.

**Table S7: Association of sociodemographic, socioeconomic factors and health status with SARS-CoV-2 seropositivity across study periods; sensitivity analysis including participants who replied to the questionnaire more than 60 days before and after their blood sample, Corona Immunitas study, Switzerland, May 2020 – September 2021**

| **Factor** | | **Period 1, n=3280**  (01/05/2020- 31/10/2020) | **Period 2, n=5248**  (01/11/2020- 15/05/2021) | **Period 3^b^, n=2840**  (16/05/2021-31/09/2021) | |
| --- | --- | --- | --- | --- | --- |
|  | | **Model 1** ^a^**, PR (95% CI)** | **Model 1** ^a^**, PR (95% CI)** | **Model 1** ^a^**, PR (95% CI)** | **Model 2** ^b^**, PR (95% CI)** |
| Sex | |  |  |  |  |
|  | Female | 1 [Reference] | 1 [Reference] | 1 [Reference] | 1 [Reference] |
|  | Male | 1.16 (0.84 - 1.61) | 1.04 (0.92 - 1.19) | 0.93 (0.89 – 0.98) | 0.97 (0.94 - 1.00) |
| Age groups | |  |  |  |  |
|  | ≥ 65 | 1 [Reference] | 1 [Reference] | 1 [Reference] | 1 [Reference] |
|  | 20-64 | 2.12 (0.99 - 4.60) | 1.18 (0.94 -1.49) | 0.85 (0.78-0.93) | 0.94 (0.89 - 0.99) |
| Children in the household | | |  |  |  |
|  | No children | 1 [Reference] | 1 [Reference] | 1 [Reference] | 1 [Reference] |
|  | One child | 0.67 (0.38 – 1.16) | 1.21 (0.98 - 1.50) | 0.90 (0.81 – 1.00) | 0.97 (0.90 - 1.04) |
|  | More than one child | 1.06 (0.69 - 1.63) | 1.12 (0.92 - 1.36) | 0.92 (0.85 -1.00) | 1.06 (0.99 - 1.12) |
| Educational level | | |  |  |  |
|  | Tertiary | 1 [Reference] | 1 [Reference] | 1 [Reference] | 1 [Reference] |
|  | Secondary | 1.67 (1.17 - 2.38) | 0.86 (0.75 - 0.98) | 0.95 (0.91 – 0.99) | 1.01 (0.98 - 1.04) |
|  | Primary | 1.41 (0.53 - 3.73) | 1.13 (0.87 - 1.46) | 0.92 (0.82 - 1.03) | 1.01 (0.93 - 1.10) |
| Household income | |  |  |  |  |
|  | > CHF 9'000 | 1 [Reference] | 1 [Reference] | 1 [Reference] | 1 [Reference] |
|  | CHF 6'000 - 9'000 | 0.66 (0.41 - 1.04) | 1.04 (0.88 - 1.23) | 0.88 (0.83 – 0.93) | 0.99 (0.95 - 1.03) |
|  | CHF 3'000 - 6'000 | 0.80 (0.50 - 1.30) | 1.23 (1.03 - 1.47) | 0.83 (0.78 - 0.88) | 0.97 (0.94 - 1.01) |
|  | < CHF 3'000 | 0.75 (0.36 - 1.55) | 0.91 (0.69 - 1.20) | 0.75 (0.68 - 0.82) | 0.94 (0.89 - 1.00) |
| Employment status | |  |  |  |  |
|  | Retired | 1 [Reference] | 1 [Reference] | 1 [Reference] | 1 [Reference] |
|  | Outside the labour force^c^ | 1.07 (0.46 – 2.51) | 0.67 (0.53 – 0.85) | 0.84 (0.72 – 0.97) | 1.00 (0.90 – 1.10) |
|  | Self employed | 1.15 (0.54 - 2.45) | 0.70 (0.51 - 0.97) | 0.78 (0.70 - 0.88) | 0.98 (0.90 - 1.06) |
|  | Employed | 0.63 (0.29 - 1.37) | 0.83 (0.65 - 1.07) | 0.84 (0.77 - 0.93) | 1.00 (0.95 - 1.06) |
| Body Mass Index | | |  |  |  |
|  | 18.5 – 24.9 | 1 [Reference] | 1 [Reference] | 1 [Reference] | 1 [Reference] |
|  | <18.5 | 0.68 (0.22 - 2.13) | 1.05 (0.70 - 1.58) | 0.99(0.86 - 1.14) | 1.04 (0.93 - 1.16) |
|  | 25 – 29.9 | 0.78 (0.54 - 1.13) | 1.12 (0.97 - 1.29) | 1.08(1.03 - 1.13) | 1.04 (1.00 - 1.07) |
|  | ≥ 30 | 0.70 (0.42 - 1.18) | 1.16 (0.95 - 1.40) | 1.12(1.04 - 1.19) | 1.04 (1.00 - 1.09) |
| Comorbiditiy score^d^ | |  |  |  |  |
|  | 0 | 1 [Reference] | 1 [Reference] | 1 [Reference] | 1 [Reference] |
|  | 1 | 1.11 (0.76 - 1.63) | 0.94 (0.82 - 1.09) | 1.08(1.03 - 1.13) | 1.01 (0.98 - 1.04) |
|  | ≥2 | 1.14 (0.68 - 1.93) | 1.22 (1.03 - 1.45) | 1.12(1.06 - 1.18) | 1.00 (0.97 - 1.03) |
| Smoking habit | | |  |  |  |
|  | Non-smoker | 1 [Reference] | 1 [Reference] | 1 [Reference] | 1 [Reference] |
|  | Smoker | 0.77 (0.48 - 1.22) | 0.83 (0.68 - 1.00) | 0.93 (0.86 - 1.00) | 0.97 (0.92 - 1.01) |
| ^a^ Model adjusted for: sex, age, educational level, body mass index, income, employment status, children in the household, comorbidity score and smoking habit  ^b^ Model additionally adjusted for vaccination status  ^c^ Outside the labour force includes participants in training/studying and not employed participants  ^d^ Comorbidity score goes from 0 to >2 and was calculated using the following possible answers: cancer; immunological diseases; cardiovascular diseases or diabetes or hypertension; respiratory diseases; allergies | | | | | |

**Table S8:** **Association of recommended preventive behaviours with SARS-CoV-2 seropositivity across study periods, sensitivity analysis including participants who reply to the questionnaire more than 60 days before and after their blood sample, Corona Immunitas study, Switzerland, May 2020 – September 2021**

| **Factor** | | **Period 1, n=2318**  (01/05/2020- 31/10/2020) | **Period 2, n=5248**  (01/11/2020- 15/05/2021) | **Period 3, n=2839**  (16/05/2021-31/09/2021) | |
| --- | --- | --- | --- | --- | --- |
|  | | **Model 1** ^a^**, PR (95% CI)** | **Model 1** ^a^**, PR (95% CI)** | **Model 1** ^a^**, PR (95% CI)** | **Model 2** ^b^**, PR (95% CI)** |
| Physical distancing during previous 7 days | |  |  |  | |
|  | Frequently | 1 [Reference] | 1 [Reference] | 1 [Reference] | 1 [Reference] |
|  | Occasionally/rarely | 1.23 (0.62 - 2.44) | 1.54 (1.21 - 1.97) | 0.81 (0.75 - 0.88) | 0.97 (0.92-1.02) |
| Staying at home during previous 7 days | |  |  |  | |
|  | Frequently | 1 [Reference] | 1 [Reference] | 1 [Reference] | 1 [Reference] |
|  | Occasionally/rarely | 1.60 (0.99 - 2.55) | 1.16 (1.00 - 1.36) | 0.94 (0.90 - 0.98) | 1.02 (0.99-1.5) |
| Wearing mask during previous 7 days | |  |  |  | |
|  | Frequently | 1 [Reference] | 1 [Reference] | 1 [Reference] | 1 [Reference] |
|  | Occasionally/rarely | 0.76 (0.48 - 1.20) | 1.03 (0.76 - 1.38) | 0.76 (0.70 - 0.84) | 0.96 (0.91-1.02) |
| Hygiene measures during previous 7 days | |  |  |  | |
|  | Frequently | 1 [Reference] | 1 [Reference] | 1 [Reference] | 1 [Reference] |
|  | Occasionally/rarely | 1.14(0.43 - 3.04) | 0.97 (0.70 - 1.35) | 0.79 (0.72 - 0 .87) | 1.01 (0.94-1.08) |
| Note: data from Ticino period 1 and data from Vaud period 1 were not included because not harmonizable with data from other sites  ^a^ Model adjusted for sex, age, educational level, body mass index, income, employment status, children in the household, comorbidity score and smoking habit  ^b^ Model additionally adjusted for vaccination status | | | | | |

**Table S9: Sensitivity analysis including 20-34 age category, Corona Immunitas study, Switzerland, May 2020 – September 2021**

| **Factor** | | **Period 1, n=3108**  (01/05/2020- 31/10/2020) | **Period 2, n=4969**  (01/11/2020- 15/05/2021) | **Period 3^b^, n=2836**  (16/05/2021-31/09/2021) | |
| --- | --- | --- | --- | --- | --- |
|  | | **Model 1** ^a^**, PR (95% CI)** | **Model 1** ^a^**, PR (95% CI)** | **Model 1** ^a^**, PR (95% CI)** | **Model 2** ^b^**, PR (95% CI)** |
| Age groups | |  |  |  |  |
|  | ≥ 65 | 1 [Reference] | 1 [Reference] | 1 [Reference] | 1 [Reference] |
|  | 35-64 | 2.04 (0.92 - 4.48) | 1.13 (0.89 - 1.44) | 0.87 (0.80-0.94) | 0.94 (0.89 - 0.99) |
|  | 20-34 | 2.87 (1.23 - 6.71) | 1.60 (1.20 - 2.12) | 0.64 (0.57-0.73) | 0.88 (0.81-0.96) |
| ^a^ Model adjusted for: sex, age, educational level, body mass index, income, employment status, children in the household, comorbidities and smoking habit  ^b^ Model additionally adjusted for vaccination status | | | | | |

#

# **Table S10: Sensitivity analysis including preventive behaviours score, Corona Immunitas study, Switzerland, May 2020 – September 2021**

| **Factor** | **Period 1, n=2151**  (01/05/2020- 31/10/2020) | **Period 2, n=4969**  (01/11/2020- 15/05/2021) | **Period 3^b^, n=2836**  (16/05/2021-31/09/2021) | |
| --- | --- | --- | --- | --- |
|  | **Model 1** ^a^**, PR (95%)** | **Model 1** ^a^**, PR (95%)** | **Model 1** ^a^**, PR (95%)** | **Model 2** ^b^**, PR (95%)** |

| Preventive behaviours score^c^ | |  |  | |
| --- | --- | --- | --- | --- |
| 0 | 1 [Reference] | 1 [Reference] | 1 [Reference] | 1 [Reference] |
| 1 | 0.77 (0.44 – 1.36) | 1.15 (0.99 – 1.35) | 0.99 (0.94 – 1.03) | 1.02 (0.99 – 1.05) |
| 2 | 0.82 (0.42 - 1.61) | 1.07 (0.76 – 1.50) | 0.92 (0.85 – 1.00) | 1.01 (0.95- 1.06) |
| 3 | 1.07 (0.40 – 2.82) | 1.23 (0.68 – 2.20) | 0.69 (0.59 – 0.81) | 0.96 (0.87 – 1.07) |
| 4 | 1.77 (0.45 – 7.01) | 1.61 (0.73 – 3.54) | 0.64 (0.50 – 0.81) | 0.99 (0.85 – 1.17) |

| ^a^ Model adjusted for: sex, age, educational level, body mass index, income, employment status, children in the household, comorbidities and smoking habit  ^b^ Model additionally adjusted for vaccination status  ^c^Preventive behaviours score goes from 0 to 4; a score=0 means that participants frequently adhered to all the preventive behaviours, a score=4 means that participants occasionally/rarely adhered to all the preventive behaviours |
| --- |
